# Ubiquitin-mediated DNA damage response is synthetic lethal with G-quadruplex stabilizer CX-5461

**DOI:** 10.1038/s41598-021-88988-w

**Published:** 2021-05-07

**Authors:** Tehmina Masud, Charles Soong, Hong Xu, Justina Biele, Saelin Bjornson, Steven McKinney, Samuel Aparicio

**Affiliations:** 1 Department of Molecular Oncology, BC Cancer Agency, 675 West 10th Avenue, Vancouver, BC V5Z 1L3 Canada; 2 Department of Pathology and Laboratory Medicine, University of British Columbia, Vancouver, BC V6T 2B5 Canada

**Keywords:** Cancer, Cell biology, Drug discovery, Diseases, Molecular medicine, Oncology

## Abstract

CX-5461 is a G-quadruplex (G4) ligand currently in trials with initial indications of clinical activity in cancers with defects in homologous recombination repair. To identify more genetic defects that could sensitize tumors to CX-5461, we tested synthetic lethality for 480 DNA repair and genome maintenance genes to CX-5461, pyridostatin (PDS), a structurally unrelated G4-specific stabilizer, and BMH-21, which binds GC-rich DNA but not G4 structures. We identified multiple members of HRD, Fanconi Anemia pathways, and POLQ, a polymerase with a helicase domain important for G4 structure resolution. Significant synthetic lethality was observed with UBE2N and RNF168, key members of the DNA damage response associated ubiquitin signaling pathway. Loss-of-function of RNF168 and UBE2N resulted in significantly lower cell survival in the presence of CX-5461 and PDS but not BMH-21. RNF168 recruitment and histone ubiquitination increased with CX-5461 treatment, and nuclear ubiquitination response frequently co-localized with G4 structures. Pharmacological inhibition of UBE2N acted synergistically with CX-5461. In conclusion, we have uncovered novel genetic vulnerabilities to CX-5461 with potential significance for patient selection in future clinical trials.

## Introduction

Folded G-quadruplex (G4) structures arise transiently at guanine-rich sequences in the DNA and RNA of multiple organisms, including microbes, yeast, plants, *C. elegans*, mouse and humans^1–9^. G4 formation is thought to play important roles in biological processes such as gene transcription, translation, telomere maintenance, DNA replication, and recombination^10–12^. Although discovered in the human genome at single-stranded 3’ overhangs in telomeres and in the promoter regions of oncogenes, recent large scale DNA sequencing-based studies on the human genome have revealed over 700,000 putative G4 sites^10,13–16^. Approximately 10,000 of these putative sites, as captured by G4 structure-specific Chip-Seq, were found to be enriched at regulatory, nucleosome-depleted regions including promoters and 5’UTRs, and in somatic copy number amplified regions^17^. G4 forming regions have also been associated with transcriptional start sites^18^.

During DNA replication and transcription, stable G4 structures may result in replication stalling and transcriptional dysregulation. Several factors are involved in the resolution of stable structures to allow for duplex refolding, replication or transcription. A number of in vitro and in vivo studies have demonstrated the roles of SF1 helicases, PIF1 and DNA2, and SF2 helicases, including RECQ (BLM and WRN) and Fe-S (BRIP1 or FANCJ and RTEL1) families, in enzymatically unwinding G4 DNA structures during replication^19–24^. G4 unfolding by BRIP1 depends on either REV1 polymerase-mediated G4 destabilization or WRN/BLM helicases^19,25^. In the absence of the factors involved in G4 resolution, genome integrity is compromised resulting in chromosomal deletions, rearrangements and mutations. Mammalian cells lacking homologous recombination (HR) factors BRCA1 and BRCA2, also demonstrate instability at G4 templates in the presence of G4 ligands such as pyridostatin, CX-3543, and CX-5461^26,27^. Additionally, G4 stabilizers activate ATM- and ATR-mediated DNA damage signaling as well as the non-homologous end-joining (NHEJ) pathway to repair the damage^27,28^.

Although DNA G4 structures were proposed as potential drug targets in 1989^29^, G4 stabilizing molecules or G4 ligands became available for clinical use only within the last few years.

CX-5461 is an orally bioavailable derivative of quarfloxin^30^. CX-5461 has at least two important activities. It stabilizes G4 structures and inhibits ribosomal RNA (rRNA) synthesis through RNA polymerase I^27^, although these two activities may not be mutually exclusive. A phase I clinical trial has established an initial human safety profile in advanced myelomas, non-Hodgkin lymphomas and acute leukemias (ACTRN12613001061729)^31^. In addition, CX-5461 has also shown a promising antitumor effect in preclinical studies against many cancer types, including MYCN-amplified neuroblastomas, ovarian and prostate cancers^32–37^. Recently, the G4 stabilizing activity of CX-5461 inducing DNA damage, in part by replication stalling, was shown to be synthetically lethal with loss of homologous recombination repair. RNA polymerase 1 activity was not required for this targeting mechanism^27^. In addition to polymerase stalling, it has recently been suggested that G4 associated DNA topoisomerase 2 (TOPII) inhibition may also result in double stranded breaks^38^. CX-5461 is currently in phase I/II clinical trial (NCT02719977) for BRCA1/2-mutated breast cancers^27^ based on this insight and has shown preliminary evidence of clinical activity^39^.

Since double stranded break induction is a key mechanism for some of the G4 stabilization drugs, a more complete understanding of DNA repair synthetic lethality beyond the known homologous recombination repair pathways could lead to expanded clinical indications. Here we address this for CX5461 and associated control compounds by screening at depth for synthetic lethal interactions with 480 genes, including core DNA repair genes. The initial screen identified many members of the HRD and FANC pathways, including REV1 and BRCA2 genes which are known for G4 structure resolution, as well as ATM, and ATR which are activated by CX-5461, confirming the sensitivity of the screen. POLQ, a gene in the alternative NHEJ pathway, was identified and validated in a number of cell line backgrounds. We also discovered that the loss of factors UBE2N (also known as UBC13) and RNF168, involved in DNA damage response (DDR)-related ubiquitin signaling, sensitizes cells to G4 stabilizers. We further demonstrated that the ubiquitination pathway was activated on cellular treatment with G4 stabilizers and that CX-5461, in combination with a UBE2N inhibitor, increased cancer cell toxicity.

## Results

### Loss of function screen of core DNA repair factors with G4 ligands

**Figure 1:**
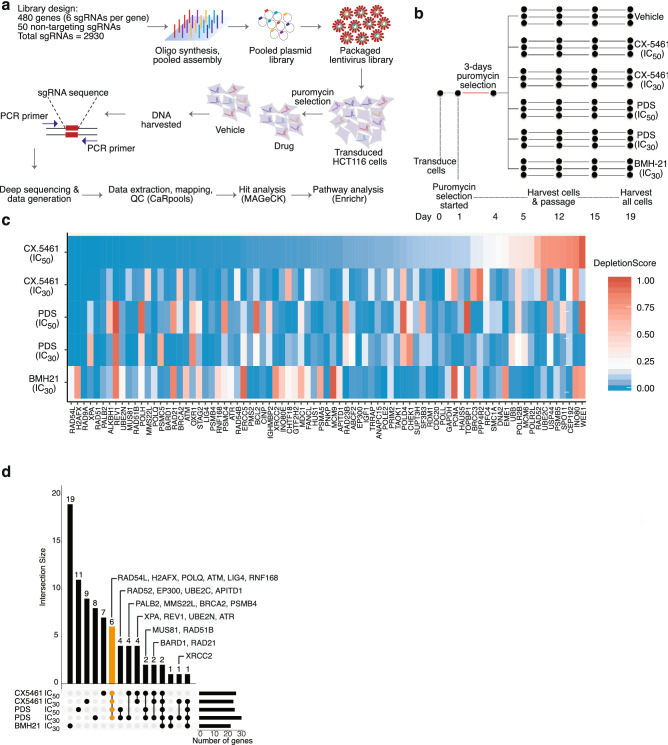
Pooled CRISPR-Cas9 screens reveal genetic determinants of sensitivity to G4 drugs. (**a**) Schematic of pooled screen. (**b**) Experimental design of drug screens. HCT116 cells were transfected with the LCV2-puro library at an MOI of 0.3. After 24 h, 0.5 µg/ml puromycin was added and the cells kept in puromycin for 3 days. On day 5 drug/vehicle treatments were started in triplicate and continued until day 19. Cells were passaged with freshly added vehicle or drugs, and harvested on days 1, 4, 5, 12, 15 and 19 to make DNA for the downstream sequencing library preparation. (**c**) Top 81 depleted genes from MAGeCK output. Each row represents the corresponding drug treatment groups. Heatmap shows the MAGeCK depletion scores of the genes. The heatmap is ordered according to the depletion scores of CX-5461 (IC_50_) group, ranking the most depleted genes on the left. To emphasize genes with the highest depletion score, the median of the color gradient (white) was shifted to 0.2. (**d**) UpSet plot showing the intersection of candidate genes undergoing negative selection with G4 stabilizers (MAGeCK *P*-values < 0.045) within each drug condition. In the bottom matrix, each row represents the set of genes identified for the indicated drug condition, and each column represents the intersection of the drug condition gene sets indicated by black dots. The histogram to the right of the matrix indicates the number of genes identified for each input drug condition. The histogram on the top shows the number of genes in each intersection set. Gene hits common in all CX-5461 and PDS conditions are shown in yellow.

In order to identify new DNA repair-associated genetic interactions with CX-5461 with high sensitivity, a pooled targeted single guide RNA (sgRNA) library was generated (Fig. 1a). This targeted library was comprised of 480 genes corresponding to DNA damage sensing, DNA repair, DNA replication, cell cycle checkpoints, chromosome segregation, and chromosome structure and remodeling as well as a set of positive control or essential genes (Table 1 and Supplementary Data)^40–43^. Since CX-5461 also inhibits RNA polymerase I complex-mediated transcription of rRNA, we included POLR1A, POLR1B, UBTF, RRN3, and TAF1A genes to represent gene expression (transcription) pathways. The library contained a total of 2930 sgRNAs with 6 sgRNAs targeting each gene and 50 non-targeting sgRNAs (NT-sgRNAs) (Supplementary Data). The quality of the pooled library was determined by next-generation sequencing which revealed that more than 99% of sgRNAs were present in the library with less than an eightfold difference in the representation of 80% of them (Supplementary information Fig. 1a) and by functional assessment of known essential genes (methods).

**Table 1:** Cellular pathways represented in the subgenomic library.

Database	Pathway	Genes
Reactome2016	DNA repair Cell cycle checkpoints Chromosome maintenance DNA Damage/telomere stress	108 105 94 58
KEGG pathway Espeseth et al	– –	21
In-house list	Gene Expression (transcription) DDR-related E3 ubiquitin ligases Others	94

To characterize the factors for which loss confers sensitivity to G4 drugs in cancer cells, we performed dropout screens in HCT116 cells with various drugs (Fig. 1b). To control for chemical scaffold-specific off-target effects, cells were treated with two G4 drugs of completely different structural classes, pyridostatin (PDS) and CX-5461, at two pre-determined inhibitory concentrations (IC) for each drug (IC_50_, the half-maximal inhibitory concentration and IC_30_). Since CX-5461 (but not PDS) also inhibits RNA polymerase I at high concentrations^27^, cells were also treated in parallel with BMH-21, an RNA polymerase I inhibitor, which binds GC rich DNA but is not a DNA G4 stabilizer at IC_30_, to identify and exclude gene-drug fitness effects resulting from RNA polymerase I inhibition. We have previously shown that BMH-21 does not induce DNA damage in epithelial cells and despite inhibiting RNA polymerase I it does not exhibit synthetic lethality with HR deficiency. The drug treatments were started in triplicates on day 5 after transduction and continued for a total of 14 days. Cells were also harvested intermittently at days 7 and 10 of the treatment, and sgRNA representation analysis was done as described for the dropout screens (Supplementary information Fig. 1).

By consolidating the most depleted genes from different treatment groups of CX-5461 and PDS, and excluding those that were shared with the top hits from the BMH21 screen, we identified the genetic vulnerabilities specific to the G4 interacting drugs. First, for each treatment condition, the effects were relatively ranked using negative depletion scores and *P*-values calculated using MAGeCK at day 19 relative to vehicle (Supplementary Data). The MAGeCK depletion scores represented the RRA (robust rank aggregation) values of the individual genes in negative selection, taking into account the performance of the 6 individual sgRNAs per gene. Top depleted genes for each condition, ranked by the depletion scores, were then consolidated together to form a final list comprising of 81 genes (Table 2). The depletion scores for each treatment condition were ordered according to the scores of CX-5461 treatment group at IC_50_ (Fig. 1c). Apart from PDS-IC_50_, many of the top depleted genes were shared between CX-5461 and PDS, but not BMH-21 (Fig. 1d.)

**Table 2:** Top depleted genes for each drug treatment.

CX-5461 (IC50)	CX-5461 (IC30)	PDS (IC50)	PDS (IC30)	BMH21 (IC30)
ALKBH1	ABCF2	APITD1	APITD1	ANAPC15
ATM	ATM	ATM	ATM	BCL2
ATR	ATR	BRCC3	BARD1	CDC20
BARD1	BARD1	CDC20	BRCA2	CEP192
BRCA2	CDC20	CHTF18	CDC20	CHEK1
H2AFX	CINP	EME1	EP300	IGHMBP2
LIG4	DNA2	EP300	FANCL	MCM6
MMS22L	ERCC5	GAPDH	H2AFX	PMS2
MUS81	H2AFX	GTF2H2	HAUS5	PNKP
OXR1	LIG4	H2AFX	INO80	POLD4
PALB2	MCM9	HUS1	INO80E	POLE2
POLH	MDC1	IGF1	LIG4	POLR2B
POLQ	POLL	LIG4	MMS22L	POLR2L
PSMB4	POLQ	MUS81	MUS81	RAD23B
PSMC4	RAD21	POLQ	PALB2	RAD51
PSMC5	RAD51	PPP4R2	PCNA	RAD9A
RAD21	RAD54L	PRIM2	PNKP	RDM1
RAD51	RAD9A	PSMA5	POLQ	RFC4
RAD51B	REV1	RAD51	PSMB4	SUPT3H
RAD54B	RNF168	RAD51B	PSMB5	TAOK1
RAD54L	SF3B3	RAD52	RAD21	TOPBP1
RAD9A	SPO11	RAD54L	RAD51	UBB
REV1	UBE2N	RAD9A	RAD51B	USP44
RNF168	XPA	RNF168	RAD52	
STAG2	XRCC2	TRRAP	RAD54L	
UBE2N		UBE2C	RAD9A	
XPA			RNF168	
			SMC1A	
			UBE2C	
			WEE1	
			XRCC2	

Candidates of interest included genes that were exclusively shared between different CX-5461 and PDS treatment groups (Fig. 1d). Hits in common with the BMH-21 treatment group, including RAD9A and RAD51, were excluded from future analysis as they likely represent genes not involved purely in G4 stabilization associated repair. We thus identified 58 genes including those known to be involved in G4 resolution, such as BRCA2 and REV1. As expected, multiple members of the HR pathway such as RAD54L, RAD52, PALB2, RAD51B exhibited negative fitness with G4 ligands. We also observed genes whose homologs were previously identified in screens performed in *C. elegans* (ATM, MUS81, POLQ)^27^. Gene hits common to all CX-5461 and PDS treatment conditions included RAD54L, H2AFX, POLQ, ATM, LIG4, and RNF168.

Next, we searched for cancer-related alterations in our top candidate genes using the cBio-Portal for Cancer Genomics database^44,45^. Most of these genes are mutated in many types of cancers (Supplementary information Fig. 3). Although all cancer types with loss of function or decreased gene expression of these genes could potentially benefit from CX-5461 therapy, we decided to focus on genes that could also serve as therapeutic targets. We searched for the druggability of the top hits from the screen using the Drug Gene Interaction Database (DGIdb) and found four genes that were predicted to interact with drugs: ATM, ATR, UBE2N, and PSMB4. The ATM/ATR pathway has been shown to be activated by CX-5461 in acute lymphoblastic leukemia cells^28^. ATR inhibition enhances CX-5461-associated apoptosis in these cells. UBE2N, along with RNF168, is involved in DNA damage response-related ubiquitin signaling, and this pathway is frequently altered in cancers (Supplementary information Fig. 3). These alterations are accompanied by an associated increase (in case of amplification) or decrease (in case of deletion) in mRNA expression.

Taken together our screen identified known genes and pathways involved in G4 stabilization as anticipated from previous mechanistic studies, confirming the biological sensitivity of the screen, as well as several additional pathway members, including notably POLQ and two key members of the ubiquitin DNA damage sensing pathway, which we further followed up.

### Validation of POLQ in mediating G4 stabilizer sensitivity

**Figure 2:**
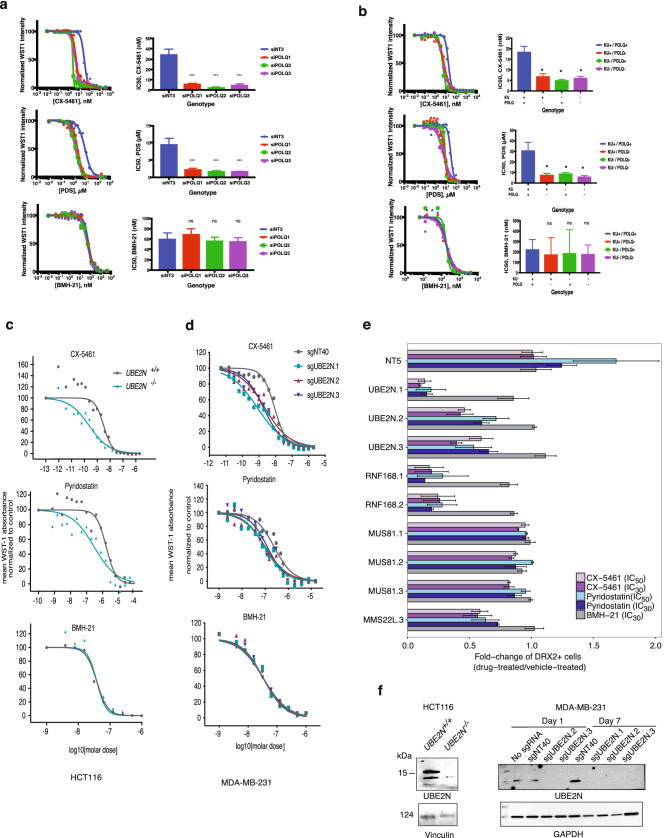
Validation of CX-5461 and Pyridostatin drug screens. (**a**) Validation of PolQ sensitivity in HCT116 cell line with siRNA.Drug-dose response of HCT116 cells transfected with individual POLQ-targeting siRNAs, followed by 7 day treatment with CX-5461, PDS, or BMH-21. Drug-dose response curves are shown on the left. Barplots on the right show the calculated IC50 values of each cell genotype (mean + 95% CI), compared to non-targeting siRNAs. ***p ≤ 0.001, analyzed by GraphPad Prism with One-way ANOVA followed by Dunnett’s multiple comparisons test, with a single pooled variance; n = 3. (**b**) Validation of PolQ sensitivity in MEF knockout cell lines. Drug-dose response of isogenic knockout MEF cells undergoing 7 day drug treatments with CX-5461, PDS, or BMH-21. Drug-dose response curves are shown on the left. Barplots on the right show the calculated IC50 values of each cell genotype (mean + 95% CI), compared to other genotypes. *p ≤ 0.05, analyzed by GraphPad Prism with One-way ANOVA followed by Dunnett’s multiple comparisons test, with a single pooled variance; n = 3. (**c**) Validation of UBE2N sensitivity in HCT116 knockout cell lines. HCT116 UBE2N-wildtype (+ / +) and -null cells (− / −) were treated with serial dilutions of CX-5461, PDS, and BMH-21 for a week followed by WST-1 assay. (**d**) MDA-MB-231 cells were transduced with lentivirus targeting the UBE2N gene or a non-targeting control (NT40) and treated with serial dilutions of CX-5461, PDS and BMH-21 for one week followed by WST-1 assay. The results are representative of at least two independent experiments, and three technical replicates for each experiment. (**e**) HCT116 cells were transduced with individual sgRNAs including a non-targeting control (NT5), three targeting UBE2N, two for RNF168, three for MUS81, one for MMS22L. Transduced and non-transduced mixed cell populations were subjected to different drug treatments for one week at indicated doses and flow analysis was performed to determine the fraction of cells expressing red fluorescence. The mean fold change relative to vehicle treatment is shown for each mixed population (bar) along with the standard error of the mean (SEM). The results are representative of at least two independent experiments, and three technical replicates for each experiment. Bootstrap analysis was performed (details in Supplementary information) and bootstrapped confidence intervals for drug effect after gene knockout are shown in Supplementary information Fig. 9. (**f**) Left panel shows UBE2N gene expression in HCT116 WT and UBE2N knockout cells by Western blot. Right panel shows Western blot results of UBE2N gene expression in all the cell lines at the start of the drug treatment (day 1) and at the end of the experiment (day 7).Uncropped blots are shown in Supplementary Fig. 12.

To evaluate the spectrum of drug sensitivity mediated by POLQ-deficiency as well as to validate the screen results, new sgRNAs were designed. As an improvement from the GeCKO v2 design, the new designs aimed to both reduce off-target effects as well as increase sgRNA stability and activity^46–48^. Sensitivities to G4 stabilizers were determined by treating WT and POLQ-null HCT116 cells with varying concentrations of CX-5461, PDS, and BMH-21 for 7 days. The cell viabilities were then assessed using the WST-1 assay. Dose-response curves were plotted to determine IC_50_ values. POLQ suppression by sgRNAs significantly decreased the IC_50_ values of CX-5461 from 30*.*27 nM to an average of 11*.*97 nM across the 5 new sgRNAs (ANOVA followed by Dunnett’s multiple comparisons tests; P-value ≤0.001; Supplementary information Fig. 4a). In a similar manner, IC_50_ values of PDS were significantly decreased from 10*.*78 to 1*.*77 µM (ANOVA followed by Dunnett’s multiple comparisons tests; *p* ≤0.001; Supplementary information Fig. 4b). In comparison, there was no difference in the sensitivity of POLQ-null cells to BMH-21 (ANOVA followed by Dunnett’s multiple comparisons tests; *p* >0.05), further supporting the fact that POLQ was mediating drug sensitivities specific to G4 stabilizers (Supplementary information Fig. 4c). In a parallel experiment, POLQ-targeting siRNAs were transfected into HCT116 cells one day prior to drug treatments. Cells treated with siRNAs then underwent the same 7-day drug treatments to assess drug sensitivities. Similar to the sgRNA experiments, siRNA-mediated POLQ suppression decreased the IC_50_ values of CX-5461 and PDS from 34*.*20 nM and 9*.*45 µM to 4*.*46 nM and 1*.*90 µM, respectively (ANOVA followed by Dunnett’s multiple comparisons tests; *p* ≤0.001; Fig. 2a). No difference in the sensitivity of POLQ-null cells to BMH-21 was detected (ANOVA followed by Dunnett’s multiple comparisons tests; *p* >0.05; Fig. 2a). While we observed differences in drug sensitivity, we sought to confirm that the sgRNAs and siRNAs were efficiently suppressing POLQ. For each drug dose response experiment with either sgRNA or siRNA treatment described above, we determined the gene expression levels by extracting the RNAs and performing qRT-PCR on the cDNAs. The relative gene expression of POLQ was assessed with respect to non-targeting controls (non-targeting sgRNA or siRNA), using GAPDH expression as endogenous controls. In both sgRNA and siRNA treatments, POLQ expression was suppressed down to 20% (Supplementary information Fig. 5a, b). The fact that sgRNA and siRNA yielded similar POLQ expression levels may not be surprising. The advantage of CRISPR/Cas9-mediated suppression lies in the permanent modification to genomic DNA, whereas siRNA-mediated suppression is always transient. However, studies have shown that in experiments spanning a short time frame, siRNA-mediated suppression is not necessarily weaker^49,50^. This could be attributed to the fact that in shorter time frames, for sgRNA-mediated disruption in the DNA, pre-existing mRNAs are continuously translated until they are turned over or depleted^51^.

To assess whether POLQ is also important in mediating G4 stabilizer sensitivities in other cell backgrounds, we performed the same drug dose response experiments in U2OS cells, and isogenic mouse embryonic fibroblasts or MEFs for the following genotypes: wild-type (WT), KU70^−/−^, POLQ^−/−^, and double knockout or DKO for KU70 and POLQ. For U2OS cells, suppression of POLQ by siRNA was confirmed by qRT-PCR (Supplementary information Fig. 5c). In U2OS cells, the IC_50_ of CX-5461 was significantly decreased from 112*.*2 to 51*.*8 nM (ANOVA followed by Dunnett’s multiple comparisons tests; *p* ≤0.001; Supplementary information Fig. 6a).

Similarly, IC_50_ of PDS was significantly decreased from 7*.*51 to 4*.*23 µM (ANOVA followed by Dunnett’s multiple comparisons tests; *p* ≤0.001; Supplementary information Fig. 6b). Again, sensitivities of POLQ-null cells to BMH-21 were unchanged (Supplementary information Fig. 6c). For MEFs, following the same drug dose response experiments, we determined that KU70 and POLQ single or double knockout cells were sensitized to both CX-5461 and PDS, but not BMH-21 (Fig. 2b). Compared to 18*.*43 nM in WT, the IC_50_ of CX-5461 in Ku70-, POLQ-, and double-knockout cells were 5*.*00 nM, 6*.*85 nM, and 6*.*15 nM, respectively (ANOVA followed by Dunnett’s multiple comparisons tests; *p* ≤0.05; Fig. 2b). Compared to 30*.*76 µM in WT, the IC_50_ of PDS in Ku70-, POLQ-, and double-knockout cells were 8*.*90 µM, 7*.*54 µM, and 5*.*94 µM, respectively (ANOVA followed by Dunnett’s multiple comparisons tests; n=3.; *p* ≤0.05; Fig. 2b). Our previous paper uncovered the increased sensitivity to CX-5461 in DNA-PK-knockout and LIG4-knockout cells^27^, which are two other members in the NHEJ pathway. The sensitivity of KU70-null cells to G4 ligands further supports the importance of NHEJ pathway in repairing CX-5461-induced DNA damage. As for POLQ, increased CX-5461 sensitivity after POLQ suppression was observed in four different cell backgrounds: *C. elegans*^27^, HCT116, U2OS, and MEF; these results suggest that POLQ-defective cells are indeed sensitive to G4 ligands.

### Re-validation of UBE2N, RNF168, MMS22L and MUS81 in growth competition assays

In order to further re-validate the results of the drug dropout screens, we selected four genes: UBE2N and RNF168 representing the DNA damage response-associated ubiquitin signaling pathway, MUS81 which is a DNA structure-specific endonuclease, and MMS22L which localizes to replication forks, as MMS22L-TONSL heterodimer, especially during replication stress^52^, to be examined individually using competitive growth assays (CGA) (Supplementary information Fig. 7). Growth competition assays provide a quantitative and robust method for determining fitness effects of drug-gene interactions. We also included an NT-sgRNA, NT5. Individual sgRNAs included those used in the library as well as new sgRNAs targeting the genes of interest (design described in methods). HCT116 cells were transduced with three individual sgRNAs representing each gene. Transduced cells, as determined by the expression of red fluorescence (DRX2), were isolated using flow cytometry and mixed with non-transduced cells. When cells were mixed in equal proportions, we observed depletion of transduced cells, irrespective of genotypes, over time by flow analysis performed on days 4, 7 and 11 after transduction, suggesting that the process of transduction affects the growth of cells (Supplementary information Fig. 8a). To adjust for different growth rates, cells were mixed in growth rate-adjusted ratios to ensure at least 40 percent of mixed populations contained transduced cells by the end of the experiment (day 11). In further validation, immunoblotting showed the efficacy of selected sgRNAs targeting UBE2N and RNF168 (Supplementary information Fig. 8c).

Mixed cell populations were treated with CX-5461, PDS and BMH-21 for 7 days at both IC_50_ and IC_30_ for PDS and CX-5461, and at IC_30_ for BMH-21. At the end of the treatment, flow analysis was performed to determine the proportion of cells expressing DRX2. The individual drug treatments alone did not result in a decrease of DRX2-expressing (DRX2+) cell populations for sgNT5 (Fig. 2e, Supplementary information Figs. 8b, 9, 10). In comparison, with UBE2N targeting, CX-5461-IC_30_ and -IC_50_ treatments consistently showed a decrease in DRX2+ cell populations (confidence interval (CI) < 0) for all sgRNAs, relative to the vehicle. PDS-IC_30_ and -IC_50_ treatments also resulted in reduced fitness for the three sgRNAs, although the magnitude of the effect was smaller than that for CX5461 treatment. In contrast, BMH-21 treatment showed no decrease at all in the fitness of DRX2+ cells. The quantitative variations observed also mirror the efficiency of sgRNAs, with sgUBE2N.1 resulting in the greatest knockout effect compared with other sgRNAs (Supplementary information Fig. 8c). For RNF168, CX-5461 (IC_30_ and IC_50_), PDS-IC_30_ and BMH-21 all showed a decrease in competitive fitness, with the effect being much stronger with CX-5461 and PDS than with BMH-21 (Fig. 2e, Supplementary information Figs. 8b and 9). For MUS81 and MMS22L.3, only CX-5461-IC_30_ showed a consistent decrease in competitive fitness effect.

Interestingly, in further experiments, we did not observe any drug-related sensitized phenotype in a MUS81-deficient HCT116 cell line (Supplementary information Fig. 11), indicating that MUS81 might be a false positive or not have a strong drug interaction. Its also possible that cells adapt rapidly to Mus81 loss and stable knockouts already have escape mechanisms. Altogether, our findings were in agreement with our screen results with the strongest re-validated gene-drug effect being associated with UBE2N and RNF168 gene targeting.

### Selective sensitivity to G4-stabilizing drugs in multiple cancer cell lines upon UBE2N depletion

Next, we characterized the dose kinetics of CX-5461, PDS and BMH-21 in human cancer cell lines upon UBE2N depletion using WST-1 cell proliferation assay. Utilizing a previously characterized UBE2N knockout (UBE2N^−/−^) HCT116 cell line^53^, we observed that UBE2N^−/−^ cells were more sensitive to CX-5461 as compared to the UBE2N^+/+^ cells (IC_50_ of 3.18 nM in UBE2N^+/+^ vs. 0.24 nM in UBE2N^−/−^; *p* <0.0001) and PDS (IC_50_ of 1*.*65 µM in UBE2N^+/+^ vs. 0*.*29 µM in UBE2N^−/−^; *p* <0.0001), and not to BMH-21 (IC_50_ of 33.82 nM in UBE2N^+/+^ vs. 43 nM in UBE2N^−/−^; *p =* 0.0974) further confirming the results of the drug screen (Fig. 2c, f, Supplementary Fig. 12 and Table 3).

Additionally, CRISPR sgRNA targeting of UBE2N in breast cancer cell line MDA-MB-231, using three different sgRNAs, revealed similar sensitivities to CX-5461 and PDS (Fig. 2d and Table 3). UBE2N-targeted cells exhibited selective sensitivity, as compared to cells containing a non-targeting sgRNA (sgNT40), to CX-5461 (IC_50_ of 8.71 nM (sgNT40) vs. 0.8 nM (sgUBE2N.1), 2.24 nM (sgUBE2N.2) and 1.85 nM (sgUBE2N.3); *p* <0.0001). PDS-treatment also conferred selective cytotoxicity upon UBE2N-targeting albeit with a milder phenotype (IC_50_ of 0*.*25 µM (sgNT40) vs. 0*.*09 µM (sgUBE2N.1), 0*.*1 µM (sgUBE2N.2) and 0*.*13 µM (sgUBE2N.3); *p* <0.0001, <0.0001 and 0.0004, respectively). In comparison, no selective cytotoxicity was observed on BMH-21 treatment in cells targeted with sgUBE2N.1 and sgUBE2N.2 as compared to those containing sgNT40 (IC_50_ of 29.32 nM (sgNT40) vs. 31.62 nM (sgUBE2N.1) and 35 nM (sgUBE2N.3); *p* >0.05). sgUBE2N.2-targeting of cells significantly increased the IC_50_ as compared to sgNT40-containing cells (IC_50_ of 38.84 nM; *p* = 0.0036). These findings suggest that the sensitivity to CX-5461 and PDS upon UBE2N depletion is not HCT116 cell line-specific.

**Table 3:** Mean IC_50_ values, 95% confidence intervals (CI) and *P*-values for CX-5461, Pyridostatin and BMH-21 dose response upon *UBE2N* gene targeting.

Cell line	Genotype	CX-5461			Pyridostatin			BMH-21		
		IC_50_ (nM)		*P*-value	IC_50_ (µM)		*P*-value	IC_50_ (nM)		*P*-value
		Best-fit	95% CI		Best-fit	95% CI		Best-fit	95% CI	
HCT116	UBE2N^+/+^ UBE2N^−/−^	3.18 0.24	2.06 to 4.92 0.16 to 0.35	– < 0.0001	1.65 0.29	1.22 to 2.29 0.17 to 0.52	– < 0.0001	33.82 43.0	29.74 to 38.72 33.25 to 56.58	– 0.0974
MDA-MB-231	sgNT40	8.71	7.24 to 10.4	–	0.25	0.19 to 0.31	–	29.32	25.88 to 33.18	–
	sgUBE2N.1	0.80	0.57 to 1.13	< 0.0001	0.09	0.07 to 0.12	< 0.0001	31.62	27.59 to 36.19	0.3959
	sgUBE2N.2	2.24	1.38 to 3.59	< 0.0001	0.10	0.08 to 0.13	< 0.0001	38.84	33.69 to 44.79	0.0036
	sgUBE2N.3	1.85	1.33 to 2.57	< 0.0001	0.13	0.09 to 0.16	0.0004	35.0	27.55 to 44.38	0.1745

### RNF168- and UBE2N-dependent histone ubiquitination at DNA damage and G4 sites after CX-5461 treatment

**Figure 3:**
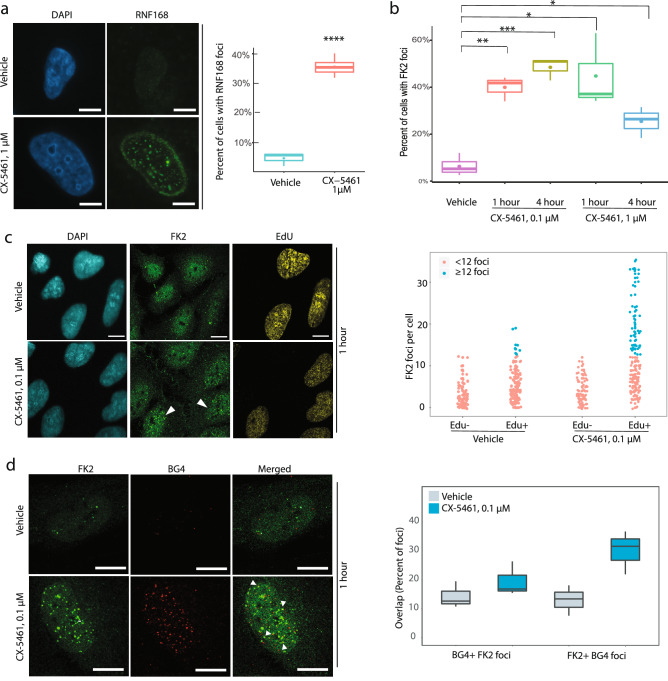
DDR-associated ubiquitin signaling is activated by CX-5461 treatment. (**a**) RNF168 foci were induced in U2OS cells after 4 h of incubation with 1 µM CX-5461. Left panel shows the images of RNF168 and DAPI staining. Right panel shows the percent of cells with RNF168 foci after CX-5461 and vehicle treatments. ****p < 0.0001 (two-tailed unpaired t-test).RNF168 foci were manually counted, and RNF168 foci positive cells are cells with foci number ≥ 3 per nucleus. (**b**) FK2 foci were induced in U2OS cells after CX-5461 treatment at indicated doses and durations of drug treatment. The box plots show the median, 25% and 75% quantiles. The mean is indicated by a dot inside the box plot. *p < 0.05, **p < 0.01, ***p < 0.001 (two-tailed unpaired t-test). (**c**) CX-5461-induced FK2 foci are enriched in S-phase of cell cycle. U2OS cells were incubated with 10 µM EdU for 20 min to label S-phase cells followed by incubation with vehicle or 0*.*1 µM CX-5461 for 1 h. Foci were visualized using confocal microscopy. The graph on the right shows the number of FK2 foci per cell in Edu- (negative) and Edu + (positive) cells. Cells with 12 or more FK2 foci are shown as blue and the cells with less than 12 FK2 foci per cell as red. The difference in the number of FK2 foci in Edu + cells is significant between CX5461 and vehicle treatments (*p* < 0.0001, TukeyHSD test). (**d**) Co-localization of FK2 foci and BG4 foci in U2OS cells after 1 h of treatment with either vehicle or CX-5461 at the indicated dose. The graph on the right shows the overlap of BG4 and FK2 foci as either percent of total FK2 foci that are also BG4 + (BG4 + FK2 foci) or percent of total BG4 foci that are also FK2 + (FK2 + BG4 foci). Scale bars in all experiments represent 10 µM. Pictures are representative of at least three independent experiments. In each experiment, at least 100 cells were counted, and the experiments were repeated more than 3 times. All the data were pooled and statistical analysis was performed on all the pooled cells. FK2 and BG4 foci per cell were counted by Spotcounting software developed by Steven Poon. The software is publicly available at github: https://github.com/shahcompbio/SpotCountingApp.

Since RNF168 and UBE2N are jointly involved in marking histones at the sites of damage, we next sought to examine the relationship between CX5461-induced DNA damage, G4 sites and histone ubiquitination as part of the DDR. As RNF168 is recruited to DNA double strand breaks (DSBs) in UBE2N/RNF8-dependent manner, we first examined the induction of RNF168 foci in U2OS cells after CX-5461 treatment using immunofluorescence (Fig. 3a). Indeed, CX-5461-treated cells showed a significant induction of RNF168 foci in approximately 35*.*46 % ± 3*.*13 % of the nuclei, as compared to 4*.*73 % ± 1*.*87 % in vehicle-treated cells (*p* <0.001). Furthermore, there was a high percentage of overlap between RNF168 foci and DNA damage marker 53BP1 foci 63*.*66 % ± 10*.*53 %. Without drug treatment, substantial overlapping was also observed (56*.*25 % ± 7*.*46 %), suggesting RNF168 activation is associated with endogenous and exogenous DNA damage (Supplementary information Fig. 14). All these results indicate that RNF168 is likely to be involved directly in the repair of CX-5461-induced DNA damage.

Next, we investigated nuclear ubiquitination since UBE2N and RNF168 promote mono- and poly-ubiquitination at sites of DNA DSBs. To determine if nuclear ubiquitin signaling is triggered in response to CX-5461-induced DNA damage, we examined ubiquitin conjugates at nuclear DNA DSBs using an antibody specific for mono- and poly-conjugated ubiquitin (FK2) and on chromatin, using immunofluorescence and Western blotting, respectively. Nuclear FK2 foci were observed in approximately 39*.*93 % ± 4*.*29 % and 48*.*47 % ± 3*.*94 % of U2OS cell nuclei after 0*.*1 µM CX-5461 treatment for 1 and 4 h, respectively, as compared to 6*.*29 % ± 3*.*29 % of vehicle-treated cells (Fig. 3b). A significant increase in the percentage of cells with nuclear FK2 foci, albeit at a lower proportion than after 0*.*1 µM CX-5461, was also observed with 1 µM CX-5461. Approximately, 44*.*77 % ± 12*.*95 % and 25*.*42 % ± 5*.*41 % of drug-treated cells demonstrated an increase in nuclear FK2 foci after 1 and 4 h, respectively, as compared to the vehicle treatment. An increase in the percentage of cells with FK2 foci was also observed with PDS treatment (Supplementary information Fig. 13a), suggesting that the DNA damage ubiquitination response is induced by multiple G4-stabilizers of different structural classes.

**Figure 4:**
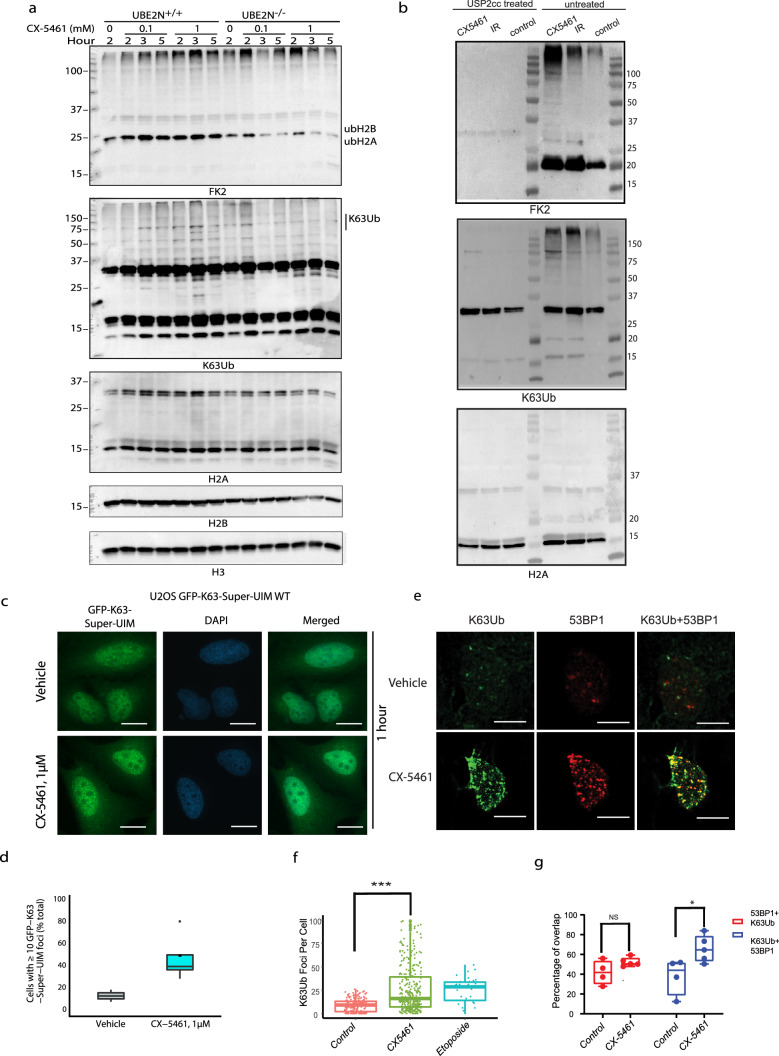
CX-5461 induces K63-linked chromatin ubiquitination. (**a**) *UBE2N*^+/+^ and *UBE2N*^−/−^ HCT116 cells were treated with either vehicle or CX-5461 at indicated doses and time periods, and chromatin fractions were extracted. Chromatin ubiquitination was examined using Western blots with antibodies against conjugated ubiquitin (FK2), K63-linked ubiquitin (K63Ub), histones H2A, H2B and H3. Molecular weight markers (KD) are shown on the left. Uncropped blots for H2A, H2B and H3 are shown in Supplementary Fig. 12. (**b**) FK2 and K63Ub signals were specifically eliminated by USP2cc. HCT116 cells were treated with either vehicle, CX-5461 at 1 µM, or IR 10 Gy for 2 h before chromatin fractions were extracted. Then chromatin proteins were equally separated into two fractions, with one fraction being treated with USP2cc and one without. Chromatin ubiquitination was examined using Western blots with antibodies against conjugated ubiquitin (FK2), K63-linked ubiquitin (K63Ub), and H2A. The experiments were repeated three times. Molecular weight markers (KDa) are shown. (**c**) U2OS cells with dox-inducible GFP-K63-Super-UIM were treated with either 1 µM CX-5461 or vehicle 24 h after doxycycline induction. Cells were fixed at indicated time and images were obtained using the same exposure time. Cells with 10 or more foci were counted using ImageJ v1.51 (https://imagej.net/ImageJ1). For each condition, at least 100 cells were examined. (**d**) Quantification of GFP-K63 foci positive cells on the images obtained in (**c**). The box plots show the median, 25% and 75% quantile, and outliers for the percentage of cells with ten or more GFP-K63 foci. The mean is indicated by a dot inside the box plot. The results are representative of the pooled data from two independent experiments. *p* = 0.0566, when the difference between CX-5461-treated group and vehicle treated group was compared (two-tailed unpaired t-test). (**e**) K63Ub foci co-localize with 53BP1 after treatment with 1 µM CX-5461 in U2OS cells. U2OS cells were treated with or without CX-5461 for 4 h, and the K63Ub foci was detected by immunofluorescence staining with an antibody specific for ubiquitin modification at K63. (**f**) Quantification of K63Ub foci per cell on the images obtained in e. The difference in the number of K63Ub foci in each cell is significant between CX-5461 and vehicle treatment (***p < 0.001, Tukey multiple comparisons of means with 95% family-wise confidence level ). Box plots show the 10–90 percentile and medium. Means are shown as “ + ”. (**g**) Quantification of the overlap of K63Ub foci and 53BP1 foci on the images obtained in (**e**). The overlap of K63Ub foci and 53BP1 foci as either percent of total 53BP1 foci within K63Ub positive foci (53BP1 + K63Ub) or percent of total K63Ub foci within 53BP1 positive foci (K63Ub + 53BP1). *p < 0.05, NS = not significant. Two-tailed student t-tests with unequal variance were performed. Foci counting in (**f**) and (**g**) was performed by SpotCounting software developed by Steven Poon. The software is publicly available at github: https://github.com/shahcompbio/SpotCountingApp. The experiments were repeated at least 3 times and each time more than 50 cells in each condition were scored. All the data were pooled and statistical analysis was performed on all the pooled cells. Scale bars in all experiments represent 10 µM.

We further analyzed RNF168/UBE2N-dependent ubiquitination at different time points on acid-extracted histones from CX-5461-treated HCT116 cells by Western blots using FK2 antibody (Fig. 4a, Supplementary Fig. 15a, c). In contrast with the focus forming assay, the high variation in Western blot signals between contrasts precluded observations of statistically significant differences when comparing the whole lane intensity of FK2 after CX-5461 treatment in *UBE2N*^+/+^ cells, versus the vehicle treatment (Fig. 4a and Supplementary Fig. 15d), in spite of an apparent trend (1.5–1.9 fold) towards an increase in FK2 intensity (see bootstrap power analysis, methods). Histone ubiquitination was suppressed in the absence of UBE2N after 3 and 5 h of treatment at 1 µM and 0*.*1 µM, indicating that UBE2N is required for ubiquitination events resulting from CX-5461-induced DNA damage (Fig. 4a, Supplementary information Fig. 15c). FK2 signal intensity in Western blots decreased dramatically after treatment with USP2cc—the catalytic domain of a deubiquitinase, indicating that the FK2 signal in Western Blot is ubiquitination specific (Fig. 4b).

To determine if conjugated ubiquitin was indeed accumulating at the sites of DNA damage following CX-5461 exposure, we examined colocalization of FK2 foci with well-characterized markers of DNA DSBs, the phosphorylated histone H2AX (*γ*H2AX) and 53BP1. *γ*H2AX is also important in the chromatin remodeling events during DNA repair. Additionally, RNF168-mediated ubiquitination of histone H2A at K15 is crucial for 53BP1 recruitment to DNA damage sites^54^. To this end, we quantified cells with co-localized FK2 and *γ*H2AX or 53BP1 foci in U2OS cells treated with CX-5461 (0*.*1 µM) for 1 h (Supplementary information Fig. 13b). Significantly higher number of *γ*H2AX and 53BP1 foci were detected in cells exposed to CX-5461 (data not shown) than the background DNA damage foci in vehicle-treated cells, as previously descibed^27^. Over 80% of FK2 foci also co-stained with *γ*H2AX after CX-5461 treatment, as compared to 25% after vehicle treatment (*p* <0.01) (Supplementary information Fig. 13b). The co-localizatin of FK2 foci and 53BP1 foci is fairly high even in vehicle-treated cells, implying that 53BP1 marked endogenous DNA damage loci are also modified by ubiquitination. Because of the high co-localization level in vehicle treated condition, we did not observe significantly different FK2 and 53BP1 co-localization events between CX-5461 and vehicle exposures (Supplementary information Fig. 13b). However, the median increase of 63.59% in FK2 foci that co-stain with 53BP1 in CX-5461-treated cells, as compared to 43% in vehicle-treated cells, may still be biologically relevant. These findings demonstrate that CX-5461 mediated ubiquitination occurs at chromosome loci marked with DNA damage foci.

We previously demonstrated that CX-5461-induced DNA damage is replication dependent^27^. To quantify the number of S-phase cells with FK2 foci, we employed 5-ethynyl-2′-deoxyuridine (EdU) incorporation into DNA during active DNA synthesis in U2OS cells. We observed that after 1 h of treatment with 0*.*1 µM CX-5461, almost all cells (99%) with FK2 foci were EdU-positive (Fig. 3c), suggesting that these cells were mostly in S-phase.

We next assessed whether conjugated ubiquitin foci formation in response to CX-5461 exposure occurs at chromosomal G4 loci (Fig. 3d). By using an engineered, G4 structure-specific antibody BG4^55^ in U2OS cells, we observed colocalization of FK2 and BG4 foci in 31.8% of CX-5461-treated cells, as compared to 13.65% of vehicle-treated cells^27^. These findings suggest that conjugated ubiquitin accumulates at not only DNA damage sites but also at G4 structures upon exposure to G4 ligands.

### UBE2N-dependent K63-linked chromatin ubiquitination in response to CX-5461

Having established that exposure to CX-5461 triggers DDR-associated UBE2N/RNF168-mediated chromatin ubiquitination, we next examined the chromatin modifications underlying the resolution of CX-5461-induced DNA damage. UBE2N and RNF8 catalyze the formation of K63-linked ubiquitin chains on linker histone H1 flanking DNA DSBS which then recruits RNF168 to the site of damage^53^. We analyzed the induction of endogenous K63-linked ubiquitination (K63Ub) after CX-5461 treatment in chromatin fractions isolated from HCT116 cells using Western blots (Fig. 4a, middle panel, Supplementary information Fig. 15b, c, and Supplementary Fig. 12). An increase in K63-linked ubiquitination was observed 2 h after drug treatment. Loss of UBE2N reduced the accumulation of K63-linked ubiquitination (Fig. 4a, Supplementary information Fig. 15c, d). Treatment with USP2cc also greatly reduced the signal intensity of K63 Ub in Western blots, indicating that the K63 Ub signal is ubiquitination specific (Fig. 4b) To extend these observations, we additionally used U2OS cells with a stably integrated doxycyclin-inducible, green fluorescent protein (GFP)-tagged tandem ubiquitin-binding entity (K63-Super UIM) which has been shown to bind specifically to K63 linkages only^53^ (Fig. 4c, d). Indeed, we observed increased GFP-K63-Super-UIM foci in 47*.*71 % ± 19*.*8 % nuclei after CX-5461 treatment as compared to 13*.*68 % ± 4*.*47 % nuclei in the vehicle treatment. However, because of data variation, the difference was not statistically significant (*p *= 0.056, two-tailed t-test). Next, utilizing an anti-ubiquitin antibody specifically targeting K63 ubiquitination in immunofluorescence assay, we also discovered that the number of k63Ub foci per cell greatly increased after CX-5461 treatment (*p *<0.001, Tukey multiple comparisons of means with 95% family-wise confidence level, Fig. 4e, f). A high percentage of overlap between K63Ub foci and DNA damage marker 53BP1 foci was observed 52*.*29 % ± 4*.*0 % (Fig. 4g), suggesting that the CX-5461 treatment-induced DNA damage is associated with UBE2N-RNF8-mediated K63-linked ubiquitination.

### Increased sensitivity to CX-5461 in response to a small-molecule inhibitor of UBE2N

**Figure 5:**
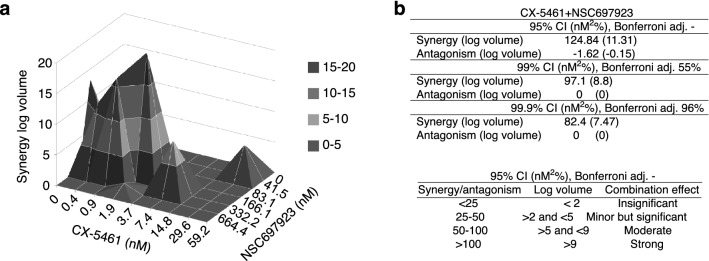
Pharmacological inhibition of UBE2N acts synergistically with CX-5461. (**a**) HCT116 cells were treated with serial dilutions of NSC697923 and CX-5461, as individual dilutions or as drug mixtures in 45 combinations, using equipotency ratios, for four days. NSC697923 was added at five doses and CX-5461 at nine. In the end, WST-1 assay was performed and results were analyzed to determine drug synergy using MacSynergyII software. The plot represents the volume of synergy produced by the drug combination. Cells with zero volume indicate additive interaction. A peak above zero is synergy, and depression below zero is antagonism. The index indicates the magnitude of the effect. (**b**) The table on the top right shows synergy or antagonism with log volumes in parentheses, at 95%, 99%, and 99.9% confidence intervals (CI) calculated with the indicated Bonferroni adjustment. The table on the bottom right shows the statistically significant cut-offs for synergy and log volumes for the combination effect. The results are representative of two independent experiments with three technical replicates for each experiment.

Although several small-molecule covalent inhibitors of UBE2N have been developed^56–58^, NSC697923 has been shown to specifically inhibit UBE2N activity via the covalent modification of the conserved active site cysteine residue critical for the catalytic activity^59^. This small-molecule inhibitor exhibits a cytotoxic effect on diffuse large B-cell lymphoma (DLBCL), neuroblastoma and malig nant melanoma cells^60–62^. The effect of simultaneous combinations of CX-5461 and NSC697923 was examined in HCT116 cells using a three-dimensional dose-response surface method^63,64^. To test a large range of drug doses, a diagonal constant ratio combination design using equipotency ratios (e.g., CX-5461 IC_50_ at 7.4 nM and NSC697923 IC_50_ at 166.1 nM) was employed so that the effect of each drug would be equal in the combination^65^. The combination of the two drugs showed synergistic response across a range of combinations in HCT116 cells treated for 4 days, with a peak at 1.9 nM of CX-5461 and 166.1 nM of NSC697923 (Fig. 5a, Supplementary Data). In addition, a strong synergy volume of 124.8 nM^2^% at 95% CI) was observed (Fig. 5b, right panel). On the other hand, the overall antagonism volume was insignificant at − 1.62 nM^2^%.

## Discussion

We have previously shown that CX5461 and other G4 binders cause selective cytotoxicity in HR-deficient cancers in part by induction of DNA breaks which cannot be repaired. This molecule is currently in early phase clinical trials to determine the therapeutic effect in patients with HR repair defects, with preliminary signs of clinical activity^39^. However the full spectrum of DNA repair deficiencies which could sensitize tumours to CX5461 and other G4 binders is not yet known. To fully saturate for genetic synthetic lethality we conducted a targeted CRISPR-Cas9 dropout screen contrasting G4 drugs, CX-5461 and PDS, with loss of function in 480 core genes involved in genome maintenance, DNA repair and replication. The inclusion of counter-screen compound BMH-21, an RNApol1 inhibitor but non-G4 binder, permitted prioritization for follow-up of hits that do not involve inhibition of RNA polymerase I, which is a known off-target effect of CX-5461 at high concentrations^27^. The sensitivity of the targeted screen was confirmed by re-identification of lethal G4 drug interaction with BRCA2, REV1, POLQ, ATM and ATR^25–27,32,66^. Among the G4 binder sensitizers we noticed convergent hits in UBE2N and RNF168, co-functional members of the histone ubiquitination DNA damage response pathway, which have not been previously reported in connection with G4 binders. The mechanism underlying selective elimination of tumor cells with compromised UBE2N- and RNF168-mediated ubiquitination, upon exposure to G4 ligands, was further characterized.

The importance of UBE2N-, RNF8- and RNF168-mediated ubiquitin signaling and post-translational histone ubiquitination in DNA DSB repair is known. UBE2N, an E2 ubiquitin-conjugating enzyme, and RNF8 and RNF168, E3 ubiquitin ligases, are recruited to DNA DSBs^67–70^. They trigger a non-proteolytic ubiquitination cascade which contributes to DNA repair and repair pathway choice by recruiting additional factors to the DSB sites. UBE2N acts together with one of its two non-catalytic variants, UBE2V1 or UBE2V2, previously known as UEV1 and MMS2, respectively^71,72^. However, UBE2V1 only participates in cytoplasmic ubiquitination. UBE2N-UBE2V2 heterodimer binds RNF8 which facilitates the formation of K63-linked ubiquitin chains on H1-type linker histones providing a binding platform for RNF168 via UDM1 domain^53,67,73–75^.

RNF168 then catalyzes the ubiquitination of H2A-type histones at K13/K15, leading to recruitment of downstream DNA repair factors, including 53BP1 which promotes NHEJ^54,76,77^. However, during S/G2 phase, RAP80 recruitment to the extended K63-linked ubiquitin chains and loss of G1-specific RIF1-dependent suppression of HR leads to recruitment of BRCA1 complex and ultimately HR^68,76,77^. We were unable to achieve sufficient knockout/knockdown of RNF8 despite multiple guide designs in different cell lines to confirm the expected role, given the involvement of RNF168 and UBE2N.

The contribution of regulatory chromatin ubiquitination to G4 structure resolution and to the repair of G4-associated DNA damage has not been investigated. To explore the underlying mechanism of lethal gene-drug interactions, we examined activation of nuclear ubiquitin signaling and subsequent chromatin ubiquitination upon exposure to G4 drugs. We showed that CX-5461 treatment resulted in significant induction of RNF168 foci in nuclei. Additionally, we observed the accumulation of conjugated ubiquitin (FK2) at DNA DSBs and of K63-linked ubiquitin chains within chromatin after CX-5461 treatment. Importantly, a significant number of CX-5461-induced FK2 foci overlapped with G4 sites marked by BG4 antibody, strongly suggesting that chromatin ubiquitination is induced in response to DNA DSBs that is associated with G4 structures stabilized by CX-5461.

RNF168 has been suggested to link H2A ubiquitination to PALB2- and RAD51-dependent HR by directly interacting with PALB2 and loading PALB2 onto damaged DNA which is redundant with BRCA1 function during HR^78–81^. Interestingly, PALB2 was one of the hits in our screens although we did not explore its role in detail. RNF168 deficiency predisposes BRCA1 heterozygous mice to cancers, and these cells are hypersensitive to PARP inhibitors^82^. Forced targeting of PALB2 to damaged chromatin bypasses RNF168 requirement for genome maintenance in BRCA1 haploinsufficiency. Previously, we showed that some BRCA1-mutated tumors that are resistant to PARP inhibitors and platinum salts are sensitive to CX-5461^27^. Inhibition of chromatin ubiquitin pathway may further increase the sensitivity of these tumors to CX-5461.

Other than regulating histone ubiquitination in DNA DSB repair, UBE2N is also involved in post-replication repair (PRR) dealing with blocks to replication fork progression^83^. Interestingly, we identified PCNA from our PDS screen, which is also a member of the PRR pathway. Likely, CX-5461-stabilized G4 structures impede DNA replication and activate PRR. PCNA mono-ubiquitination promotes TLS polymerase to bypass replication blocks with errors^84^, while UBE2N mediated poly-ubiquitination of PCNA directs an error-free lesion bypass. The detailed mechanism of how UBE2N is involved in bypassing stabilized G4 structures during replication needs further investigation.

In addition to ubiquitin mediated DDR, we validated loss of function in POLQ as synthetic lethal with CX-5461 treatment. POLQ is not essential for normal cell survival, but is over-expressed in about 70% of breast cancers regardless of HR status^85,86^. Previous studies with non-medicinal structure G4 binders have hinted at a role for polQ/TMEJ in G4 mediated damage responses, here we show that the clinical stage molecule reflects this activity. The combination of POLQ inhibitors and G4 binders could thus be an attractive treatment strategy to complement PARP inhibitor treatment. POLQ mediated MMEJ pathway has been recognized as an important pathway for DSB repair. Recently, the role of POLQ in replication stress and fork reversal has been explored. Our results suggest that POLQ might also be involved in replication bypass through G4 regions in the genome.

Recently a genome-wide shRNA screen using PDS and PhenDC3, which are not drug like molecules but are both G4 ligands, identified several genes that when depleted enhanced cell death with these G4 ligands^87^. In spite of the differences between shRNA and sgRNA/CRISPR loss of function screens, we also identified some of the genes described in the shRNA screens including BRCA2, PALB2 and POLQ. Other overlapping genes included WEE1, PCNA and PSMC4, which although significant hits within a single treatment condition with PDS or CX-5461 in our screens, were nonetheless excluded from our final priority list because of the stringent requirement to be common to at least two treatment conditions. The non-overlapping findings from these two independent studies highlights the importance of using multiple approaches to identify a comprehensive list of gene interactions.

Another recent paper analyzed CX-5461 response in *C. elegans*^88^. A chemical genetic screen was performed in *C. elegans* and homologs of human genes that are sensitive to CX-5461 were identified, but different drug sensitivity profiles between *C. elegans* and human were discovered. For example, NHEJ deficiency is sensitive to CX-5461 in human cells but not in *C. elegans*, indicating distinct drug response to G4 stabilizers in different species.

Collectively, through focused screening and re-validation, we have identified additional genetic vulnerabilities to G4 stabilizers in human cells, including POLQ as well as genes involved in DNA replication, DNA damage repair, checkpoint, and DDR-associated ubiquitination. Our results suggest that G4 stabilizer drugs could be effective for cancers with a broad range of genome instability and these results potentially expand biomarker driven patient inclusion and combination therapy with G4 stabilizer drugs.

## Methods

### Plasmids

The lentiCRISPRv2 was a gift from Feng Zhang (Addgene plasmid #52961)^89^. A fluorophore-containing variant of lentiCRISPRv2 was generated by replacing the puromycin resistance marker with the DsRed-Express2 (DRX2) fluorescence marker (LCV2-DRX2). Gel extraction with the QIAEXII Gel Extraction Kit (Qiagen) was performed on the linearized 13 kb lentiCRISPRv2 vector following a BamHI and PmeI restriction digest to remove the puromycin marker, along with downstream WPRE and UTR elements. The DRX2 insert was prepared by PCR-amplifying a 1.6 kb oligo fragment (gBlock, IDT) which included DRX2 sequence as well as the lost WPRE and UTR sequences. Joining of the DRX2 insert and the cut vector was performed using NEBuilder HiFi DNA Assembly (NEB), as per manufacturer’s instructions. After transforming the cloning reaction into Stellar chemically competent cells (Clontech) and plating on Amp resistant LB Agar plates, the final clone was selected via a 4-step screening and validation process involving colony PCR, SacII-BamHI restriction digest, Sanger sequencing, and confirmation of DRX2 expression via transient transfection into HEK293T cells.

### Cell lines and cell culture

HCT116 and MDA-MB-231 cell lines were ordered from ATCC. UBE2N-deficient HCT116 cells were a kind gift from Dr. Chunaram Choudhary (Novo Nordisk Foundation Center for Protein Research, University of Copenhagen). Dox-inducible GFP-K63-Super-UIM U2OS cells were a kind gift from Dr. Niels Mailand (Novo Nordisk Foundation Center for Protein Research, University of Copenhagen). MEF (mouse embryonic fibroblast) isogenic lines (WT, POLQ-null, KU70-null, and DKO) were kind gifts from Dr. Dale A. Ramsden^90^. MUS81 knockout HCT116 cells were kind gifts from Dr. Kiyoshi Miyagawahiyama^91^. HCT116 and U2OS cells were cultured in McCoy’s media supplemented with 10% fetal bovine serum (FBS), HEK293T and MDA-MB-231 cells in DMEM containing 10% FBS. All cells were cultured at 37 °C with 5% CO2. All cell lines were tested for mycoplasma.

### Drugs

CX-5461 (Selleckchem, cat# S2684) and BMH-21 (eMolecules) were prepared in 50 mM NaH_2_PO_4_, as described previously^27^. Pyridostatin trifluoroacetate (Sigma-Aldrich, cat# SML0678) was prepared in water, NSC697923 (Sigma-Aldrich, cat# SML0618) in DMSO, and puromycin (ThermoFisher, cat# A1113803) in water.

### Sub-genomic library sgRNA oligonucleotide design and synthesis

20mer oligonucleotide sequences representing sgRNAs were selected from Human GeCKO v2 pooled library^89^. sgRNA designs missing from the GeCKO library for DNA2, TERF2, DBF4, and TRRAP genes were added^92^. DNA oligonucleotides were synthesized as a pool using a B3 synthesizer (CustomArrayInc) platform, PCR amplified using ArrayF and ArrayR primers and Phusion HS Flex (NEB) and size selected using 1% agarose gel as described previously^89,93^. Pooled oligos were cloned into lentiCRISPR v2-puromycin plasmid, digested with *BsmbI*, at 42:1 insert-to-vector ratio, using Gibson assembly^93^. Gibson reaction was diluted fourfold to transform 25 µl of Lucigen Endura electrocompetent cells. 1 ml of transformation was grown in 100 ml of LB-ampicillin at 37 °C overnight and plasmid DNA was extracted using Endofree Plasmid Maxi Kit (Qiagen).

### Library lentivirus generation and titration

HEK293T cells were transfected, at 80% confluency, with 20 µg sgRNA plasmid pool, 10 µg pMD2.G-VSVG and 15 µg psPAX2 using TransITLT1 reagent and reduced serum OptiMEM media in ten 15 cm^2^ tissue culture dishes. 24 h later, media was replaced with collection media (DMEM, 10% FBS and 1% BSA). Media containing virus was collected 48 and 72 h later, pooled and filtered through 0*.*45 µm PVDF membrane. Virus was then concentrated 240× by ultracentrifugation at 24,000 RPM for 90 min and was re-suspended in virus collection media for 30 min on shaker at room temperature. Aliquots were stored at − 80 °C. In order to determine virus titers to transduce HCT116 cells at an MOI of 0.3, 1 million cells were plated per well into a 12-well plate. Fivefold dilutions of the virus were added to each well (0*.*000 64 µL to 50 µL) along with a no transduction control. After 24 h of incubation with the virus, cells in each well were split 1:10 into duplicate wells, one with 0*.*5 µg puromycin and other with no puromycin. After three days, cell counts were determined. Survival fraction was determined by dividing the cell count from the replicate with puromycin by cell counts from the replicate without puromycin. Viral titer was determined using (survival fraction × number of cells per well × 1000/volume of virus per well).

### Dropout screens

For dropout screens, 30 million HCT116 cells were plated at a density of 1 million cells per well in 12-well plates and transduced with 30 µL LCV2-puro library at an MOI of 0.3. After 24 h, cells were pooled and plated in 150 cm^2^ plates with fresh media containing µg/ml puromycin. Cells were pooled after 3 or 6 days of puromycin selection and cultured until day 19 in triplicates. Cells were passaged at regular intervals on days 4, 7, 10, 13 and 16. At each passage, 2 to 3 million cells were plated to maintain a representation of ∼ 700 to 1000 cells per sgRNA. Cells harvested at each passage and on day 19 were used to make DNA for sequencing library preparation. For drug screens, 50 million cells were transduced with 50 µL of the virus as described above followed by 3 days of puromycin selection. On day 5 after transduction, drug/vehicle treatments were started in triplicates and continued until day 19. Fresh growth media with drugs was added at the time of splitting cells on days 12 and 15 post-transduction. Drugs were added at the following concentrations: vehicle NaH_2_PO_4_, CX-5461 23 nM (IC_30_), CX-5461 33 nM (IC_50_), PDS 7*.*73 µM (IC_30_), PDS 11*.*92 µM (IC_50_) and BMH-21 59 nM (IC_30_). In parallel, WT and BRCA2-null HCT116 cell lines were treated with similar doses to determine sensitivity. Cells were harvested on days 1, 4, 5, 12, 15 and 19 to make DNA for downstream sequencing library preparation.

### Sequencing library preparation and next generation sequencing

1 ng of pooled sgRNA plasmid library was PCR amplified using a multiplex PCR with 0*.*3 µM mix of nine forward primers with stagger (v2NGS-F1 to v2NGS-F9) and one reverse primer v2NGS-R10, and 1x Platinum Multiplex PCR master mix (Life Technologies, cat#4464269) in a total of 100 µl reaction. The PCR conditions were: 95 °C 2 min; 20 cycles of 95 °C 30 s, 60 °C 90 s, 72 °C 60 s; 72 °C for 10 min. PCR products were checked on agarose gel for the presence of the 218 bp band. 5 µl of PCR products were cleaned using Exosap-IT (ThermoFisher Scientific, cat#78201.1.ML). Purified PCR products were barcoded using Illumina Nextera barcodes and Roche FastStart HiFi PCR reagents (cat#04738292001). Barcoded PCR products were purified on E-gel. Library quality analysis was done using Agilent Bioanalyzer. The library was sequenced on MiSeq. For dropout screens, genomic DNA was extracted from frozen cell pellets using Blood and Cell Culture DNA kit 500G (Qiagen). 10 µg of genomic DNA (representing approximately 455× coverage per sgRNA) for each sample was PCR amplified and barcoded as described above. Barcoded PCR products were purified on E-gel or on SPRIselect beads (Beckman Coulter). Pilot dropout libraries were screened on MiSeq. Drug screen libraries were sequenced on NextSeq using NextSeq High Output 75v2, 75 cycles, single-end with 40% of phiX.

### Screen data processing and analysis

Unique sgRNA sequences were extracted from raw FASTQ files and mapped to the reference library FASTA index using CaRpools v0.83 and Bowtie2 v2.2.9 with parameters to not allow any mismatch^94,95^. The sgRNA read count files generated by CaR-pools were used to perform hit analysis and generate data statistics using MAGeCK v0.5.4 with default parameters^96^. For downstream analysis including gene set enrichment and overlap between GQ-drugs, candidate genes from each treatment group (CX-5461 IC_50_ and IC_30_, PDS IC_50_ and IC_30_, and BMH-21 IC_30_) with a *P*-value of <0.045 in the MAGeCK gene rankings were combined into an 81-gene list.

### Library performance during screening

To assess library performance we conducted gene dropout screens without any G4 drugs selection to characterize the following: (1) optimal duration of puromycin selection, (2) changes in the abundance of NT-sgRNAs, (3) essential gene depletion over time, and (4) optimal length for drug screens. To this end, we performed two independent and longitudinal screens in HCT116 cells (Supplementary information Fig. 1b). Cells were transduced at a low MOI of 0.3 and subjected to puromycin selection for 3 or 6 days. A fraction of cells was harvested every three days until day 19 for sgRNA representation analysis by next-generation sequencing. sgRNA abundance and statistical analyses were performed using CaRpools and MAGeCK^94,96^.

When compared with sgRNA abundance on day 1 (pre-puromycin selection), we did not observe any remarkable difference in gene depletion on day 19 between populations treated with puromycin for 3 days or 6-days (Supplementary information Fig. 1c; Pearson *R* = 0*.*87), thus prompting us to apply only 3 days of puromycin selection for the drug screens. In addition, the abundance of NT-sgRNAs did not change as compared to the initial pool irrespective of the duration of puromycin selection (Supplementary information Fig. 1c), and in independent dropout screens (Supplementary information Fig. 1d). In comparison, gene depletion (*P*-value <0.05) was observed after 3 days of puromycin selection (Supplementary information Fig. 1e). By day 19, over 10% of the genes for cell populations undergoing 3 days of puromycin selection showed significant dropout with reference to either day 1 (pre-selection) (Supplementary information Fig. 1e) or day 4 (post-selection) (data not shown). The top depleted genes included POLR2I, GAPDH and ANAPC5. Of the top 10% of depleted genes in our screens, almost 92% of the genes were fitness genes, with 38 out of 48 genes previously described as core fitness genes and 6 as HCT116 cell line-dependent fitness genes (Supplementary Table 1)^97^.

Together, these results show that the subgenomic dropout screens were able to detect essential gene dropout, with high sensitivity and reproducibility, without a significant perturbation in the NT-sgRNA pools, thus providing a platform to perform focused functional genetic screens with drugs.

During comparative screening with different drugs we assessed the performance of individual sgRNAs in the top depleted genes of interest (Supplementary information Fig. 2). It was evident that not all sgRNAs targeting the same gene were depleted in a similar manner. As an example, for LIG4, we observed that two of the sgRNAs led to the enrichment of the respective cell populations across all treatment groups, including BMH-21. MAGeCK assessed the relative enrichment or depletion of individual sgRNAs. For LIG4, MAGeCK determined that 2 of the sgRNAs were enriched, with a positive fold-change, while the other 4 were depleted. The inefficient cutting of sgRNAs has been cited by other groups^98–100^, illustrating the importance of methods such as MAGeCK to first measure the performance of individual sgRNAs before deriving a gene-based ranking.

### Subcloning, lentivirus production and titration for individual sgRNAs

Individual sgRNAs were designed using the Deskgen Cloud CRISPR Design Software (Desktop Genetics). sgRNA cloning into LCV2-DRX2 vector was performed as previously described^89,93^. Transformation was performed by adding 2 µl ligated product into 50 µl Stellar Chemically Competent Cells (Takara) as per manufacturer’s instructions. Up to 100 µl of transformed cells in S.O.C. Medium (ThermoFisher) were plated onto LB Agar plates containing 100 mg/ml Ampicillin and grown overnight at 37 °C. The following afternoon, multiple colonies from each plate of cloned sgRNA plasmids were inoculated and grown overnight at 37 °C in 1 ml LB broth with 100 mg/ml Ampicillin. Plasmid DNA was extracted from 800 µl bacteria culture using Qiagen Plasmid Miniprep Kit (Qiagen) or Monarch Plasmid Miniprep Kit (NEB), as per manufacturer’s instructions. Plasmid samples were sent for Sanger sequencing validation, and the correct clones were expanded, by growing the remaining 200 µl bacteria culture in 200 ml LB Broth overnight, at 37 °C with shaking. Plasmid DNAs were then extracted using the PureLink HiPure Plasmid Maxiprep Kit (ThermoFisher). Lentivirus was generated as described above. To titrate lentiviruses, cells of interest were plated at 50,000–200,000 cells per well into a 24-well plate. Starting from 10 µl, fivefold dilutions of the virus were added into 250 µl of media (up to 8 dilutions). After 24 h, media were replaced with fresh media without virus. Seventy-two hours after viral transduction, flow cytometry analyses were performed using FlowJo to determine the percentage of cells expressing DRX2. Virus titration curves were generated by plotting the fluorophore-expressing fraction against the dilution factor. From the data points on the exponential part of the curve, viral titer was calculated by using (% positive cells × # cells per well × 1000/volume of virus per well).

### Competitive growth assays (CGA)

LCV2-DRX2 vectors with individual sgRNAs were produced as described above. HCT116 cells were transduced in 24-well plates at a density of 150,000 cells per well with the virus at an MOI of 0.1–0.3. Three days after transduction, cells expressing DRX2 were flow sorted on an Aria Fusion or Aria III and mixed with non-transduced cells. First, the ratio of transduced to non-transduced cells was determined to allow the untreated mixtures to contain at least 40-50% transduced cells at the end of the assay by monitoring the proportion of DRX2+ cells 4 and 7 days after transduction using a Fortessa flow cytometer. The sgRNA MMS22L.2 was excluded from the final analysis because of the low proportion of DRX2+ cells in the mixed cell population. Growth-adjusted cell mixtures were split and plated in triplicate wells to be treated with drugs or vehicle as in the drug screens. Cells were harvested 7 days after drug treatment and analyzed by flow analysis to determine the fraction of cells expressing DRX2. The ratio of the mean percentage of DRX2+ cells in drug-treated and vehicle-treated cell populations was calculated to determine the relative fold-change in the mixed cell populations for each sgRNA of interest. Results were analyzed using R. Each experiment was repeated at least twice with three technical replicates per experiment.

### Statistical analysis for CGA

Bootstrapping was performed in a blocked fashion, to reflect the conditions of the experiment. For an experiment involving *N*_*a*_ assays, *N*_*a*_ assay numbers were randomly selected with replacement (outer bootstrap block). Within the *N*_*a*_ randomly selected assays, *N*_*?*_ replicate values were then randomly selected with replacement (inner bootstrap block). The resultant data set was of the same form as the original, with the same number of assays and replicate observations within each assay. 400,000 blocked bootstrap replicate data sets were constructed and the average proportion of cells surviving calculated in the logistic space. After transforming the averages back to the raw proportion space, differences between vehicle and drug conditions were calculated. From the distribution of the 400,000 bootstrapped differences, confidence intervals were determined. Confidence intervals of 95%, 99%, 99.9% and 99.99% (corresponding to type I error rates 0.05, 0.01, 0.001, 0.0001 respectively) were determined. With 5 drug conditions to compare to the vehicle, across 10 sgRNA conditions, 50 results are being assessed. A Bonferroni adjustment for 50 comparisons means the greatest difference must show a significance level of 0.05/50 = 0.001 for a false discovery rate (FDR) of 5% or 0.01/50 = 0.0002 for FDR = 1%.

### Drug dose response analysis

Cells were plated at low densities in 96-well plates (HCT116 cells at 300 cells/well, MDA-MB-231 at 300/well) in three or four replicates. 24 h later serial dilutions of drugs were added using three or four replicates per treatment. The drug media was refreshed every 3 or 4 days. At the indicated time points, cells were assayed with WST-1 reagent (Roche, cat#CELLPRO-RO) on the plate reader. The results were analyzed using GraphPad Prism. The means for drug-treated replicates were divided by that of the vehicle-treated populations for each genotype. IC50 values were determined by fitting a four-parameter logistic curve (variable slope model) to the data.

### Immunoblotting

For chromatin ubiquitination experiments, cells were resuspended in Triton Extraction Buffer (TEB) at a concentration of 10^7^ cells/mL and lysed on ice for 10 min on a horizontal shaker at 90 rpm. Cells were then washed in TEB and resuspended in 0.2N HCl at 4 × 10^7^ cells/mL before extracting histones overnight at 4 °C. After centrifugation, the supernatant was saved and neutralized with 1/10th the volume of 2M NaOH. For each sample, 10^6^ cells were boiled in 1X Laemmli buffer and 2-mercaptoethanol at a 1:10 ratio for 10 min at 90 °C before loading in each well of SDS-PAGE. For all other Western blots, cells were boiled in 1X Laemmli buffer for 10 min at 90 °C and protein was quantified using the Pierce 660 nm Protein Assay (ThermoFisher, cat#22660). 10 µg protein was loaded in each well of a 12% SDS-PAGE and transferred to nitrocellulose or PDMS membrane at 100 V for 1.5 h or at 30 V overnight. Primary antibodies were incubated at the concentrations listed in Table 3 as per manufacturer’s instructions. After incubating with the appropriate secondary antibodies (Goat anti-mouse, Dako cat#P0447 and Abcam cat#ab97040; goat anti-rabbit, Abcam cat#ab6721; mouse anti-goat, Santa Cruz Biotechnology cat#sc-2354) at 1:10000 or 1:5000 in 5% non-fat dried milk-TBST for 1 h, membranes were visualized with Immobilon Western Chemiluminescent HRP Substrate (MilliporeSigma, cat#WBKL20500) and imaged with the ImageQuant LAS 4000 (GE Healthcare) using the ImageQuant TL software.

### Statistical analysis for FK2 and K63Ub immunoblots

Increasing levels of H3 were associated with decreasing levels of FK2 density measurements (Total FK2 (whole lane)), so H3 did not appear appropriate as a loading control. Total H2A measured via 2 bands at 15 kDa and 2 bands measured at 30 kDa (presumably dimers of H2A) provided a loading control positively associated with FK2 total. With two data points per experimental condition, independent linear fits within condition were not possible as all fits would have been degenerate, leaving no degrees of freedom for error estimation. Thus the simplifying assumption of equal slopes within all treatment conditions was made. Initial analysis on the raw western blot photo density data yielded estimates with confidence intervals (CI) extending to negative density values. Transforming the photo density values via base 2 logarithms removed the lower bound of zero, yielding an unconstrained data range from minus to plus infinity. Regression model fit diagnostics showed reasonable Gaussian behaviour for the log2 transformed data, with estimates and CIs well removed from data boundaries. Treatment condition differences in the log realm correspond to fold-change estimates back in the raw scale, facilitating interpretation of differences as compared to differences expressed in arbitrary densitometry values. With limited data available, basic linear model fits were obtained. Insufficient data was available to perform e.g. linear mixed effects models. All conditions were estimated as fixed effects in an ANCOVA model (FK2 total *∼* Total.H2A + Genotype * Treatment) with total H2A providing the ANCOVA covariate to allow for loading control adjustment. The model fit to the log2 transformed data yielded the following ANOVA table:

Analysis of Variance Table

Response: log2FK2 total

**Table:** 

	Df	Sum Sq	Mean Sq	F-value	Pr(> F)	
log2H2A	1	3.5421	3.5421	20.8650	0.0005277	***
Genotype	1	1.2704	1.2704	7.4837	0.0169976	*
Trt	6	0.2182	0.0364	0.2143	0.9655807	.
Genotype:Trt	6	1.0795	0.1799	1.0598	0.4332235	.
Residuals	13	2.2069	0.1698	.		

Significance codes: 0 *** 0.001 ** 0.01 * 0.05 . 0.1 .. 1

The positive association of log2(FK2 total) with log2(Total H2A) showed a *P*-value of 0.00052, indicating that the ANCOVA adjustment was important and necessary. The confidence intervals in Fig. 4b show that ubiquitination levels in the CRISPR knockout UBE2N^−/−^ line were generally lower than those in the wild type line, and this is reflected by the Genotype *P*-value 0.017 in the ANOVA table. The differences associated with differing treatment conditions show wide CIs, all crossing the null hypothesis value for no treatment difference (Fold-change = 1.0 on the raw scale, or zero difference in intercept values on the log2 scale). Thus there is no statistical evidence of a difference in ubiquitination levels due to treatment conditions relative to the wild type vehicle control condition. The same statistical analysis was applied to quantify the K63-linked ubiquitination signal in Western blots.

### Immunofluorescence

For RNF168 foci, U2OS cells were treated with CSK buffer (100 mM NaCl, 300 mM sucrose, 3 mM MgCl, 10 mM PIPES (pH 6.8), with proteinase inhibitors) for 4 min followed by fixation with 2% paraformaldehyde in TBS (50 mM Tris-HCl, pH7.5, 150 mM NaCl) for 20 min, and methanol for 1 min. After blocking in 3% BSA and 0.2% Tween-20 in TBS, RNF168 antibody (Table 3) was incubated overnight followed by incubation with secondary antibody for 1 h. For K63Ub foci staining, U2OS cells were treated with CSK buffer with supplements (DTT 1mM, proteinase inhibitors, phosphotase inhibitors and deubiquitination inhibitor NEM 10 mM) for 5 min on ice, then washed with TBS with supplements for 2 times, before fixation with 2% paraformaldehyde for 20 min. Cells were further treated with cold methanol for 1 min. After blocking in 3% BSA and 0.2% Tween-20 in TBS for 1 h, K63Ub antibody (Sigma 05-1308, clone Apu3) was incubated at 100 times dilution overnight followed by incubation with secondary antibody at 1000 dilution for 1 h. For colocalization, after K63 staining with primary and secondary antibodies, 53BP1 staining was performed as previously described^27^. Immunofluorescence of BG4, 53BP1 and *γ*-H2AX was described previously^27^. To visualize FK2 foci, U2OS cells grown on glass cover slides were fixed with cold methanol for 10 min and then with acetone for 30 s. Cells were permeabilized with 0.5% Triton X-100 for 25 min, then blocked for 1 h with 3% BSA and 0.2% Tween-20 in TBS. FK2 antibody (Table 3) was incubated at 1:10,000 dilution overnight. Mouse Alexa 488 secondary antibody was diluted at 1:5000 and incubated for 1 h. Because of background foci, damage foci positive cells were defined as *γ*-H2AX foci ≥ 5, 53BP1 foci ≥ 3, RNF168 foci ≥ 3, FK2 foci ≥ 10. At least 100 cells were counted for each condition, and at least two independent experiments were performed. *P*-value was determined by two-tailed unpaired t-test for the difference of means. For FK2 foci analysis in case of CX-5461 1 µM, Welch’s two-tailed test was used to determine significance because of unequal variances. DNA damage foci per cell and foci overlap were counted by Spotcounting software developed by Steven Poon, which is publicly available at github: https://github.com/shahcompbio/SpotCountingApp. For K63-linked ubiquitination, 30,000 U2OS cells with GFP-K63-Super-UIM were grown on coverslips and induced by doxycycline as described previously^53^. Drugs or vehicle were added 24 h after induction and cells were fixed in 4% paraformaldehyde for 15 min followed by permeabilization with 0.2% Triton X-100. Coverslips were mounted on glass slides with DAPI and images were obtained using an upright Colibri LED microscope. All images were taken using the same exposure time. Cell quantification was performed using ImageJ. For each condition, at least 100 cells were examined with a threshold of 10 or more foci per cell. Two independent experiments were performed. *P*-value was determined by two-tailed unpaired t-test for the difference of means.

### Drug synergy

HCT116 cells were plated in 96 well plates at a density of 800 cells per well. Next day, serial dilutions of NSC697923 and CX-5461 were prepared and drug mixtures in 45 combinations were added to cells. NSC697923 was added at five doses: IC_50_ (166.1 nM, IC_50_x2 (332.2 nM), IC_50_x4 (664.4 nM), IC_50_x0.5 (83.05 nM) and IC_50_x0.25 (41.53 nM).CX-5461 was added at nine doses: IC_50_ (7.4 nM), IC_50_x2 (14.8 nM), IC_50_x4 (29.6 nM), IC_50_x8 (59.2 nM), IC_50_x0.5 (3.7 nM), IC_50_x0.25 (1.85 nM), IC_50_x0.125 (0.93 nM), IC_50_x0.0625 (0.46 nM) and IC_50_x0.03125 (0.23 nM). All individual drug doses and no-drug controls were added in parallel. After four days of drug treatment, a WST-1 assay was performed and results were analyzed using MacSynergyII software.

## Supplementary Information

Supplementary Information 1.
