# Supplementary material for: Ubiquitin-mediated DNA damage response is synthetic lethal with G-quadruplex stabilizer CX-5461
Source: Sci Rep. 2021 May 7;11:9812. doi: 10.1038/s41598-021-88988-w (PMC8105411; doi:10.1038/s41598-021-88988-w)
Supplement: Supplementary file 1 — Supplementary Information 1. [file 41598_2021_88988_MOESM1_ESM.pdf]

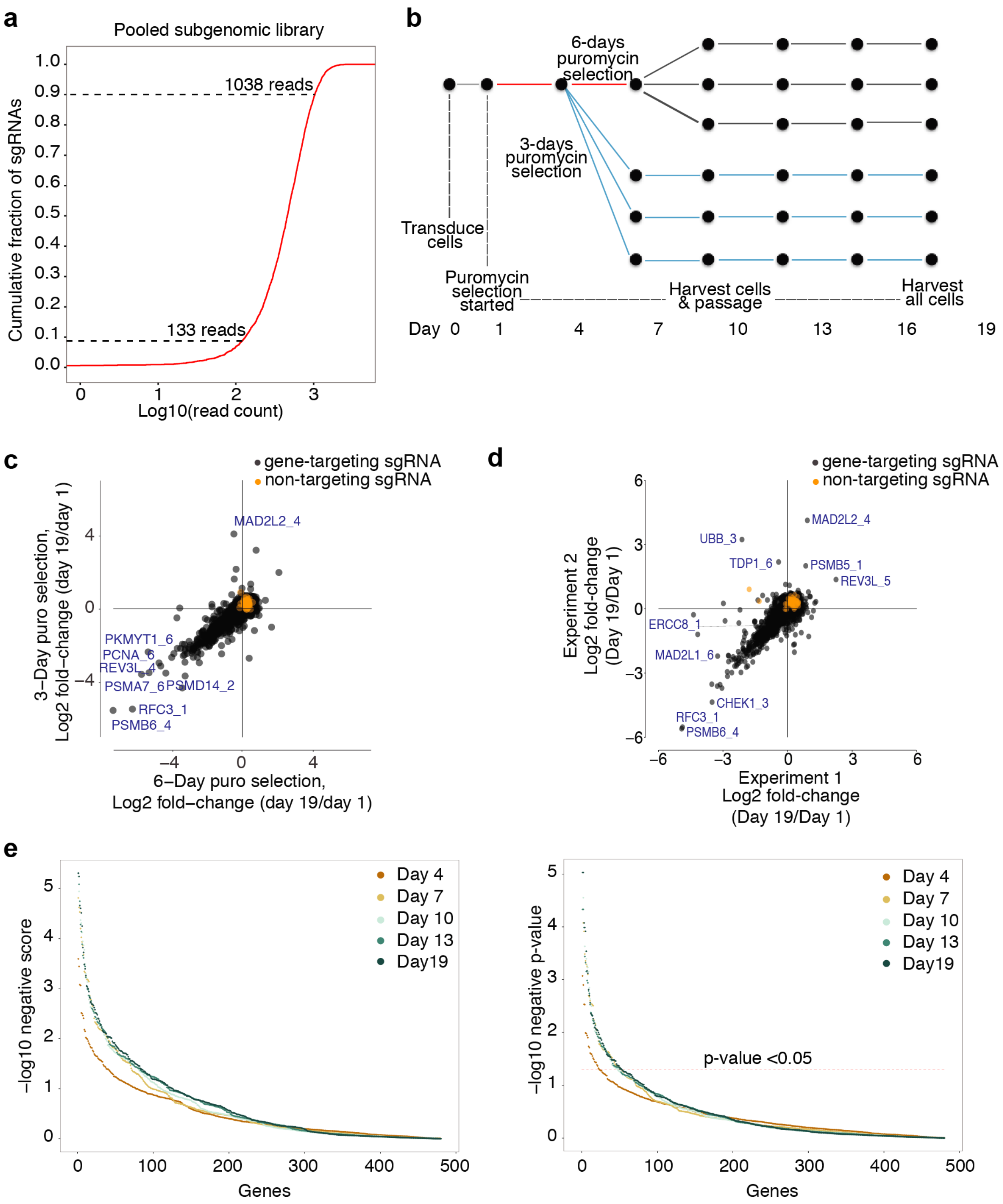

Supplementary Figure 1: Quality control analysis of pooled subgenomic library and dropout genetic screens data.

Supplementary Figure 1: Quality control analysis of pooled subgenomic library and dropout genetic screens data.

(a) Cumulative distribution of sequencing reads for all the sgRNAs in the pooled plasmid library.

(b) Experimental design of dropout genetic screens.

(c) Scatter plot showing $\log_2$ fold-change in normalized sgRNA counts on day 19 w.r.t. day 1 after 3 days of puromycin selection versus that after 6 days of puromycin selection. sgRNA counts represent means of three replicates. Non-targeting sgRNAs are shown in yellow and gene-targeting sgRNAs in black. sgRNA names showing greater than 4 or less than -4 $\log_2$ fold-change are highlighted in blue.

(d) Scatter plot showing $\log_2$ fold-change in normalized sgRNA counts on day 19 w.r.t. day 1 in two independent screens with 3 days of puromycin selection. sgRNA counts represent means of three replicates. Non-targeting sgRNAs are shown in yellow and gene-targeting sgRNAs in black. Some sgRNA names showing greater than 2 or less than -3 $\log_2$ fold-change are highlighted in blue.

(e) -$\log_{10}$(MAGeCK score) and -$\log_{10}$(p-value) distributions. For all the genes in the screen data (from the two independent screens with 3-days puromycin selection), the -$\log_{10}$(MAGeCK score) (left panel) and -$\log_{10}$(p-values) (right panel) as determined by MAGeCK are plotted for days 4, 7, 10, 13 and 19 w.r.t. day 1. Non-targeting sgRNAs were excluded from the analysis.

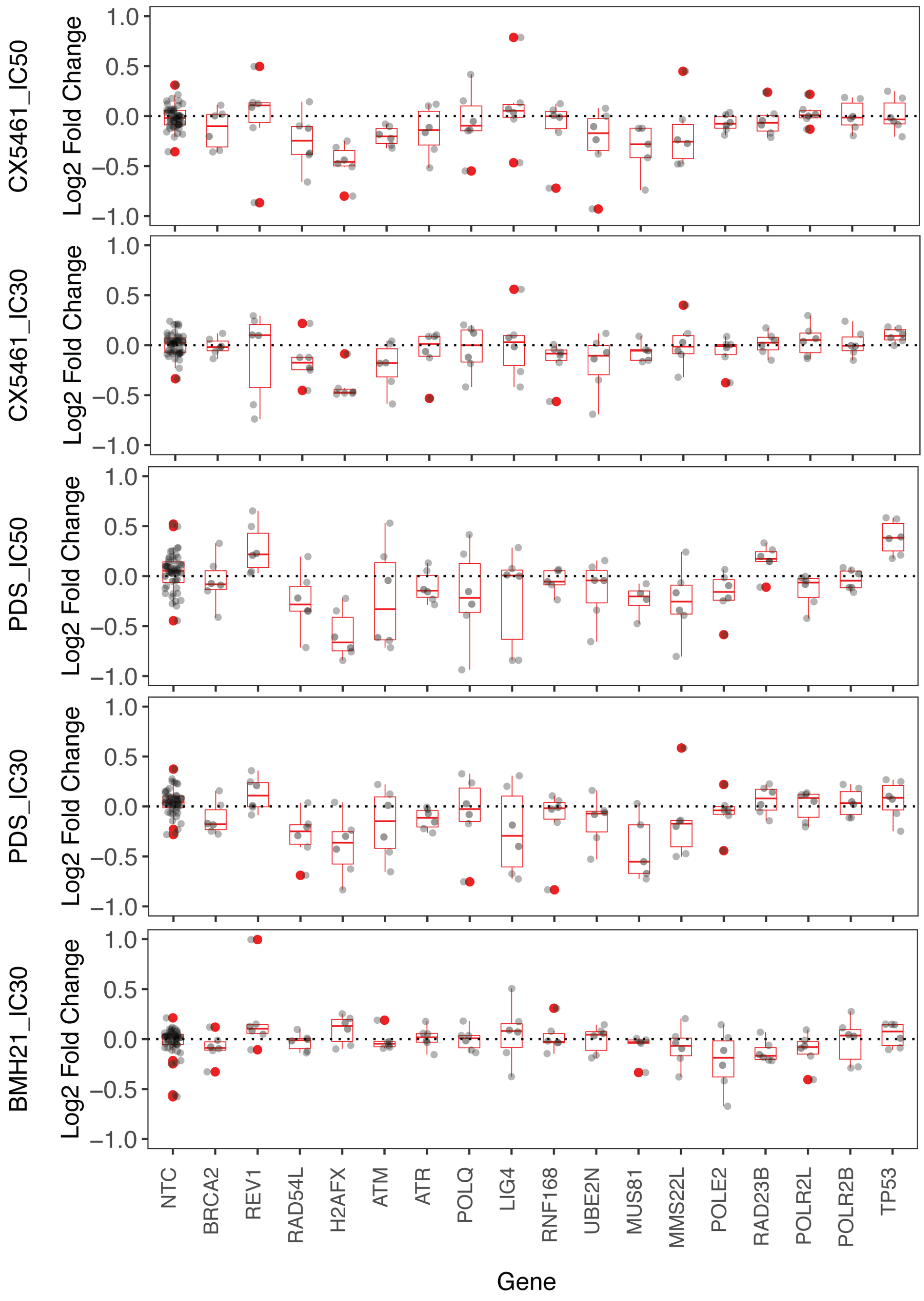

Supplementary Figure 2: Individual sgRNA fold-changes of top depleted genes.

Supplementary Figure 2: Individual sgRNA fold-changes of top depleted genes. Means of the $\log_2$ fold-change of individual sgRNAs from the drug screen, compared with non-targeting control (NTC), following 14 day treatment with CX-5461 (IC_{50} or IC_{30}), PDS (IC_{50} or IC_{30}), or BMH-21 (IC_{30}). Dots show the $\log_2$ fold-change of individual sgRNAs (6 per gene for gene-targeting; 50 sgRNAs total for non-targeting). Boxplot shows the distribution of the sgRNAs combined, with median shown by horizontal lines. sgRNAs with readcounts less than 30 were filtered. sgRNAs recognized to be outliers are shown as separate circular dots.

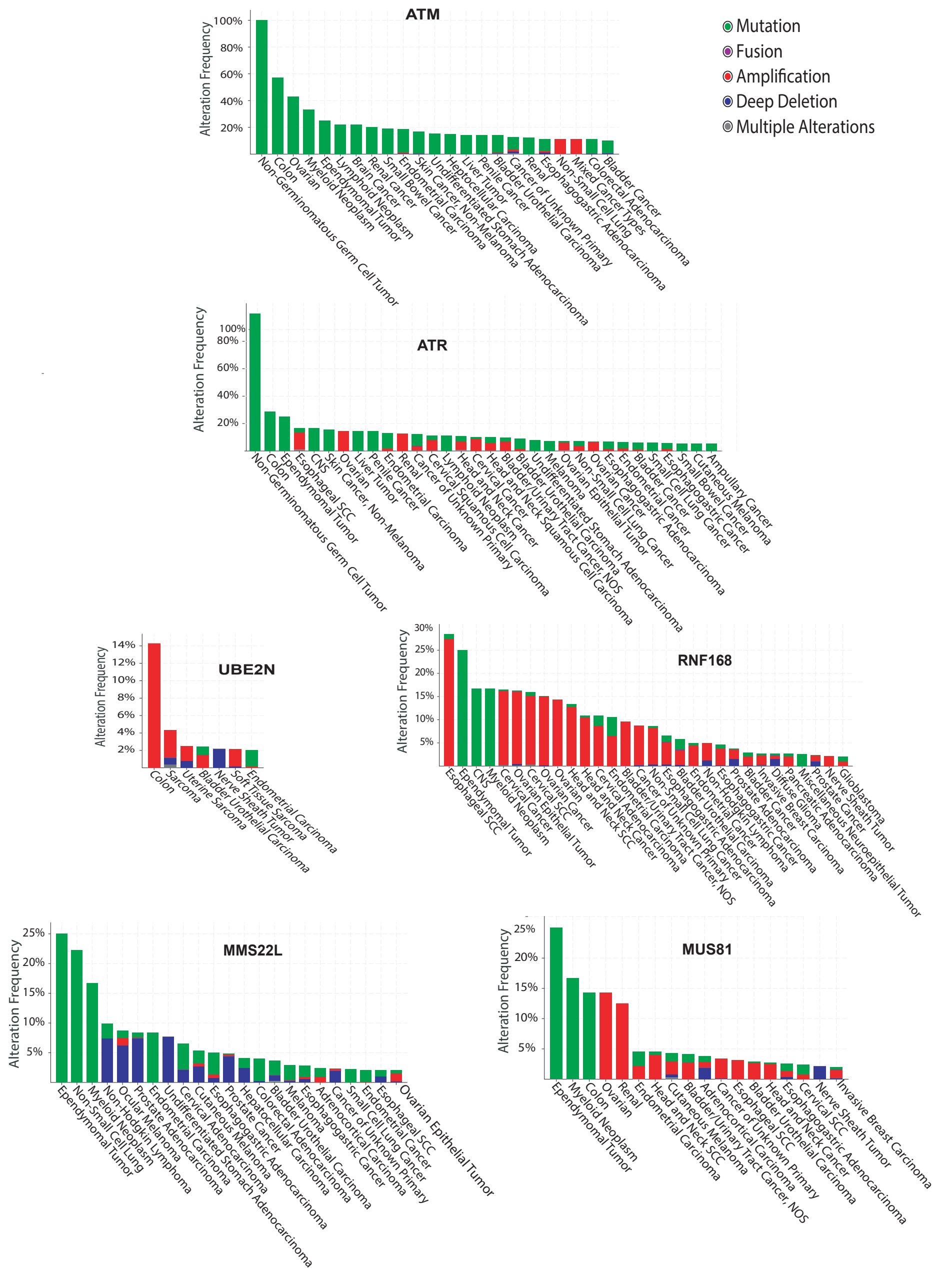

Supplementary Figure 3: Genetic alterations in top gene hits in different cancers types. Genetic alterations in genes in different cancers with a minimum of 10% (ATM), 5% (ATR) and 2% (UBE2N, RNF168, MUS81, MMS22L) of altered cases in each cancer type were determined using CBioPortal database. SCC=Squamous cell carcinoma

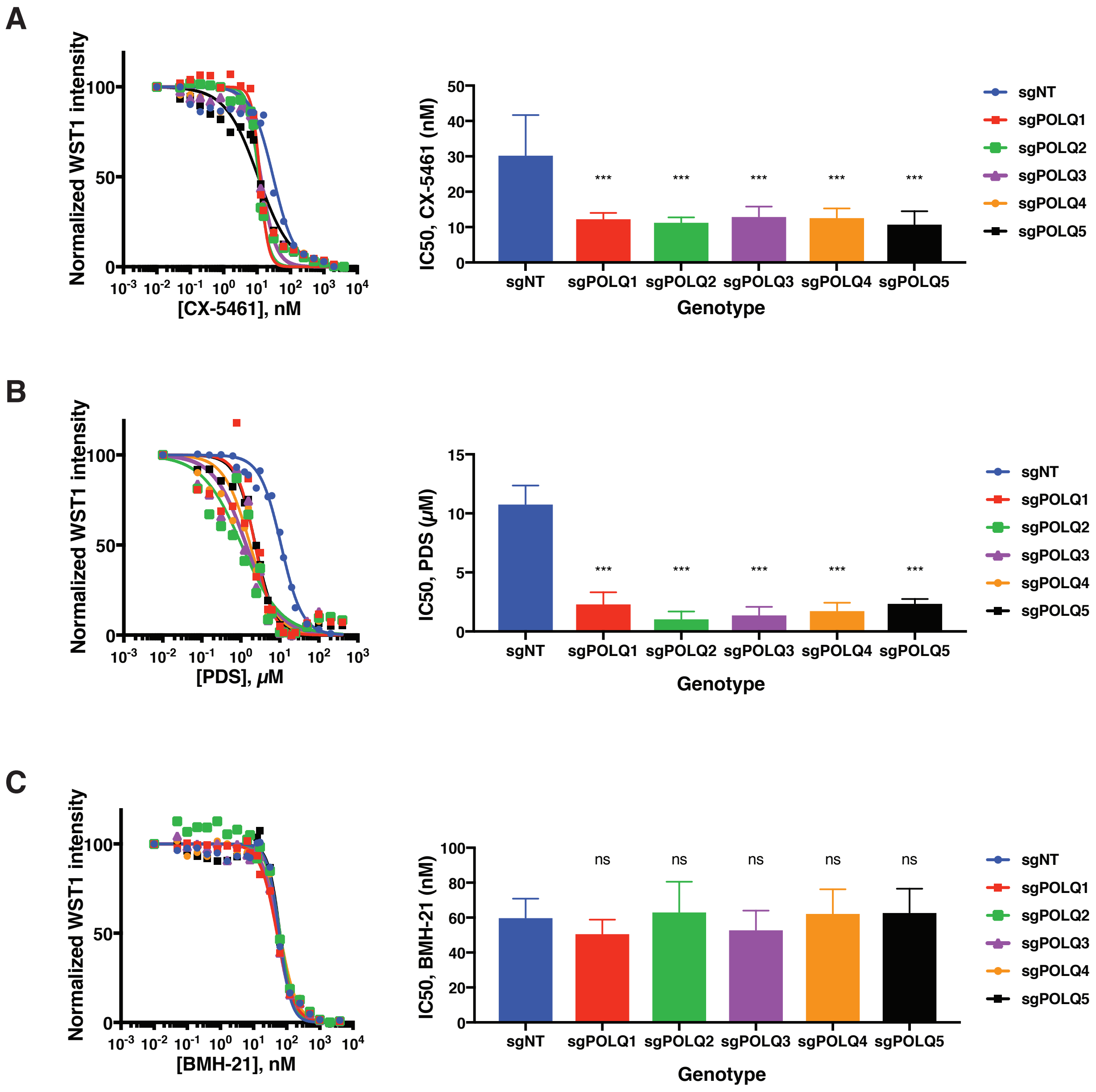

Supplementary Figure 4: CX-5461 and PDS sensitivities are replicated in targeted POLQ-depletion sgRNA experiments. (a,b,c) Drug-dose response of HCT116 cells transduced with individual POLQ-targeting sgRNAs, followed by 7 day treatment with CX-5461 (**a**), PDS (**b**), or BMH-21 (**c**). Drug-dose response curves are shown on the left. Barplots on the right show the calculated IC50 values of each cell genotype (mean + 95% CI), compared to non-targeting sgRNAs. ***p $\leq$0.001, One-way ANOVA followed by Dunnett's multiple comparison tests; n=3.

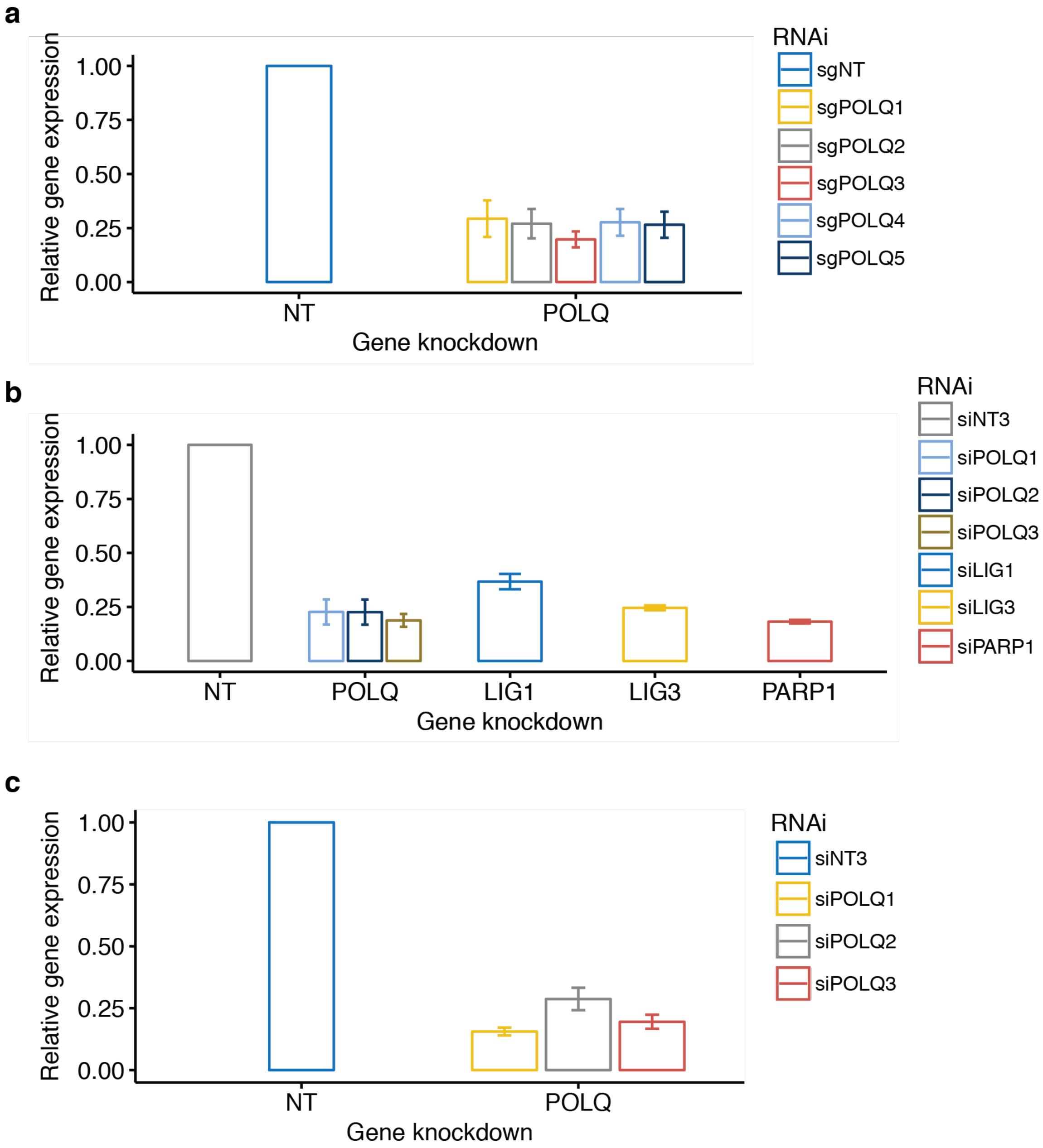

Supplementary Figure 5: siRNAs and sgRNAs are equally efficient at suppression of POLQ and MMEJ gene expressions. **(a)** POLQ gene expression following lentiviral transduction in HCT116 with sgRNAs and selection by flow cytometry. **(b)** POLQ, LIG1, LIG3, and PARP1 gene expression following siRNA transfection in HCT116. **(c)** POLQ gene expression following siRNA transfection in U2OS cells. Gene expressions are normalized to Non-targeting sgRNAs or siRNAs (NT), with respect to endogenous GAPDH expressions. n=3.

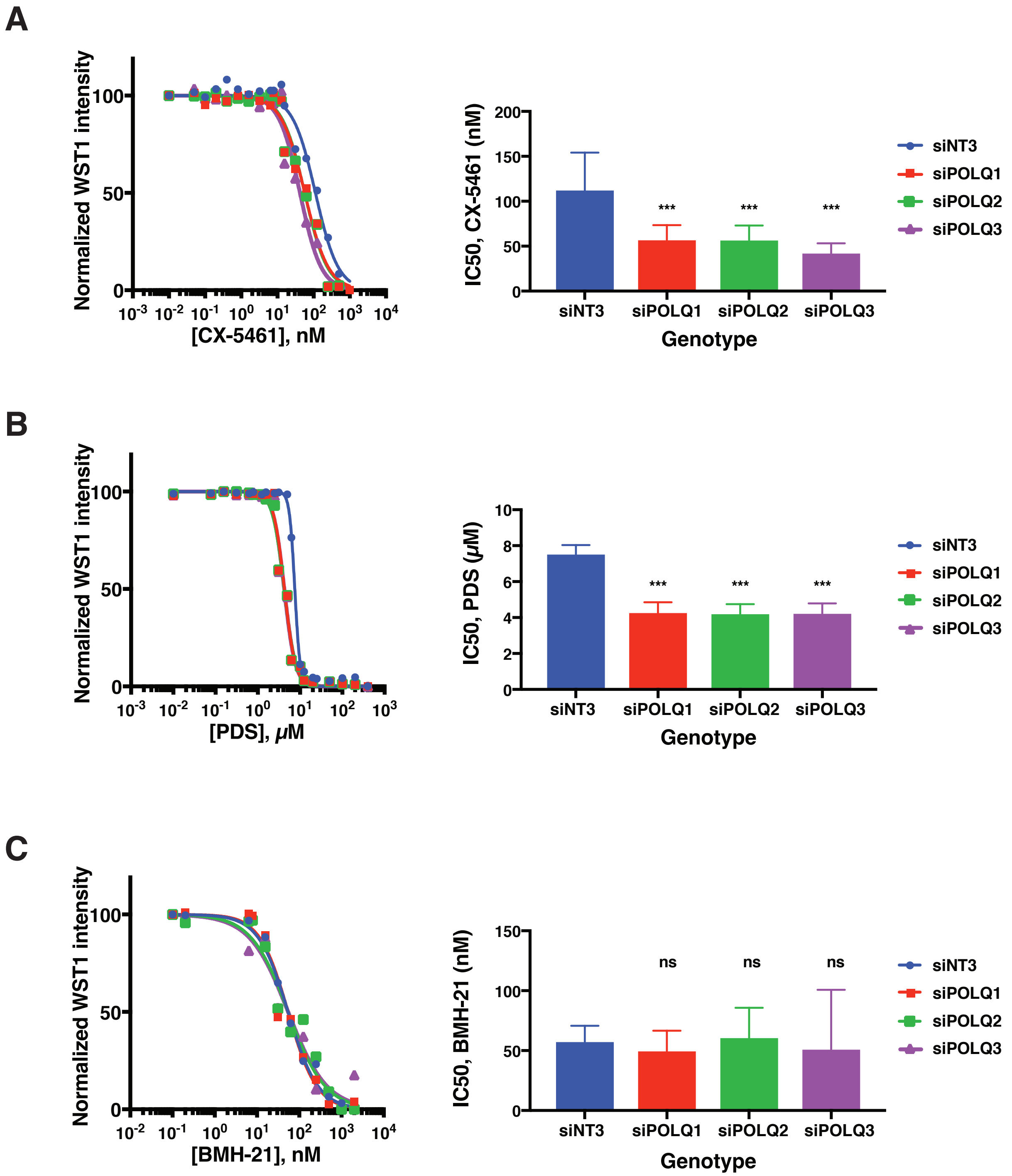

Supplementary Figure 6: CX-5461 and PDS sensitivities are replicated in POLQ-deficient U2OS cells.

Supplementary Figure 6: CX-5461 and PDS sensitivities are replicated in POLQ-deficient U2OS cells. (a,b,c) Drug-dose response of U2OS cells transfected with individual POLQ-targeting siRNAs, followed by 7 day treatment with CX-5461 (**a**), PDS (**b**), or BMH-21 (**c**). Drug-dose response curves are shown on the left. Barplots on the right show the calculated IC50 values of each cell genotype (mean + 95% CI), compared to non-targeting siRNAs. ***$p \leq 0.001$, One-way ANOVA followed by Dunnett's multiple comparison tests; n=3.

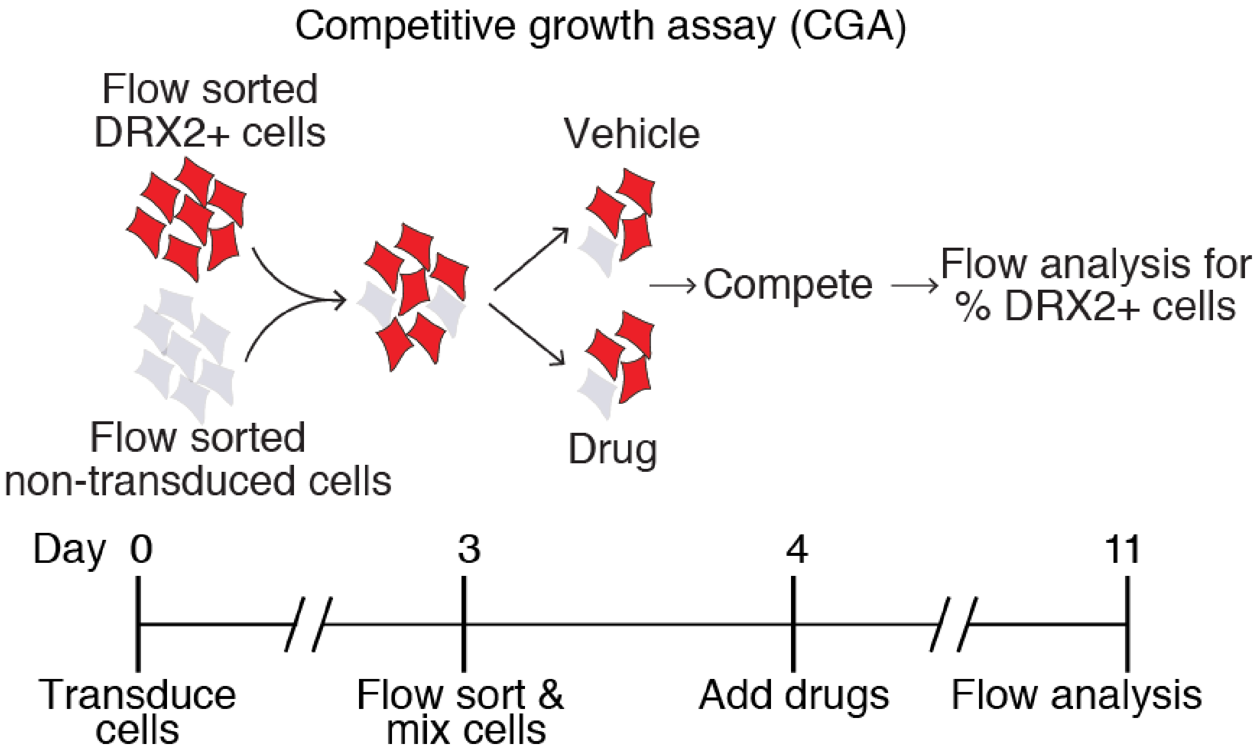

Supplementary Figure 7: Outline of competitive growth assays (CGA).

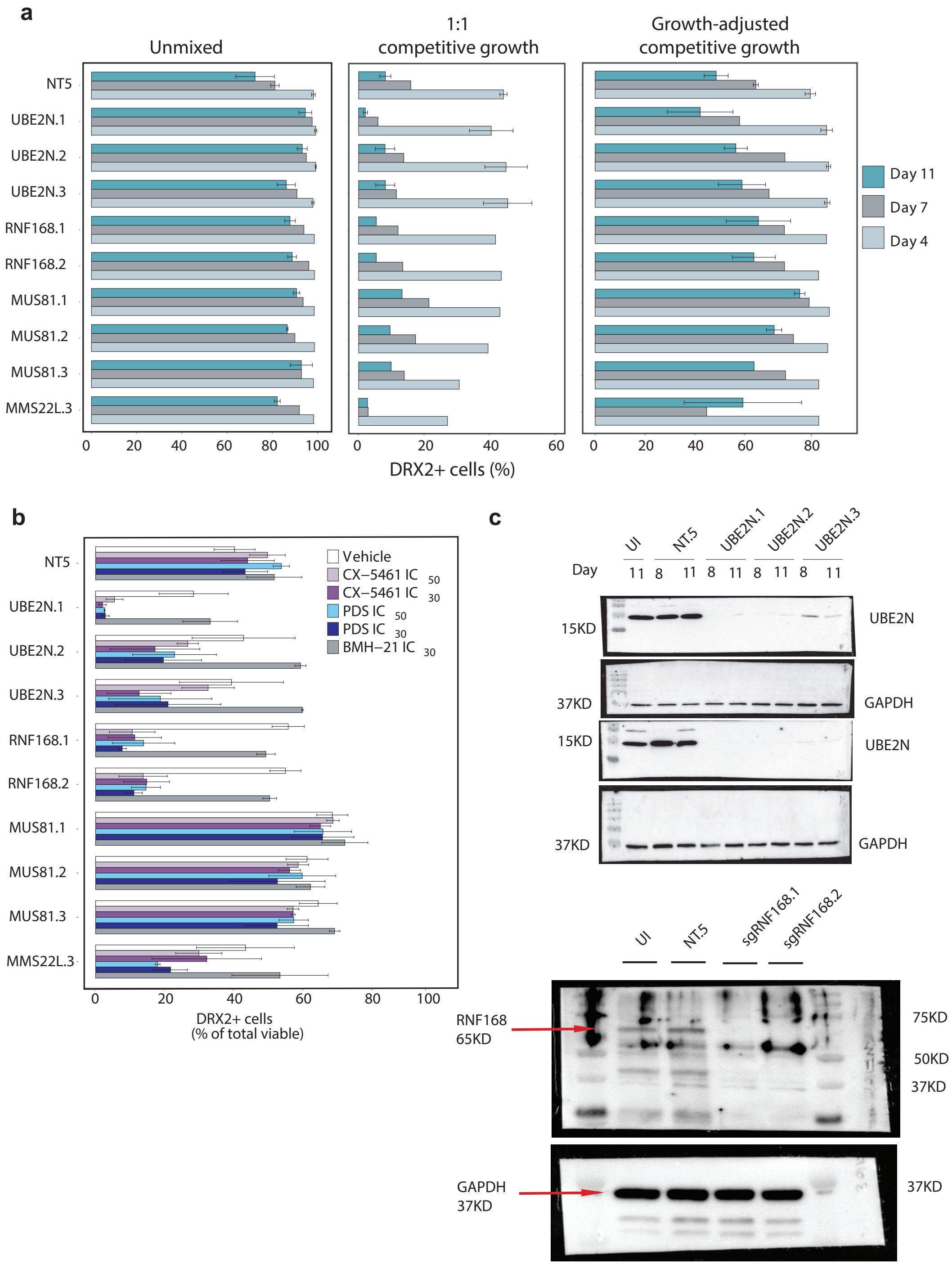

Supplementary Figure 8: Validation of drug screens in HCT116 cells.

Supplementary Figure 8: HCT116 cells were transduced with individual sgRNAs including a non-targeting control (NT5), three targeting UBE2N, two for RNF168, three for MUS81, two for MMS22L. Flow analysis was performed on transduced cell populations at indicated days after transduction to determine the fraction of cells expressing red fluorescence in **(a)**, unmixed transduced cells as well as cells mixed at 1:1 or growth-adjusted ratios, and **(b)**, mixed transduced and non-transduced cell populations subjected to vehicle, CX-5461, PDS and BMH-21 treatments for one week at indicated doses. The mean for each treatment condition is shown (bar) for each mixed population with standard error of means (SEM). **(c)** The Western blots show UBE2N, RNF168 and GAPDH (input) gene expression in the uninfected (UI) or targeted cells at the indicated days. RNF168 was knocked out with sgRNA for 8 days before Western blotting.

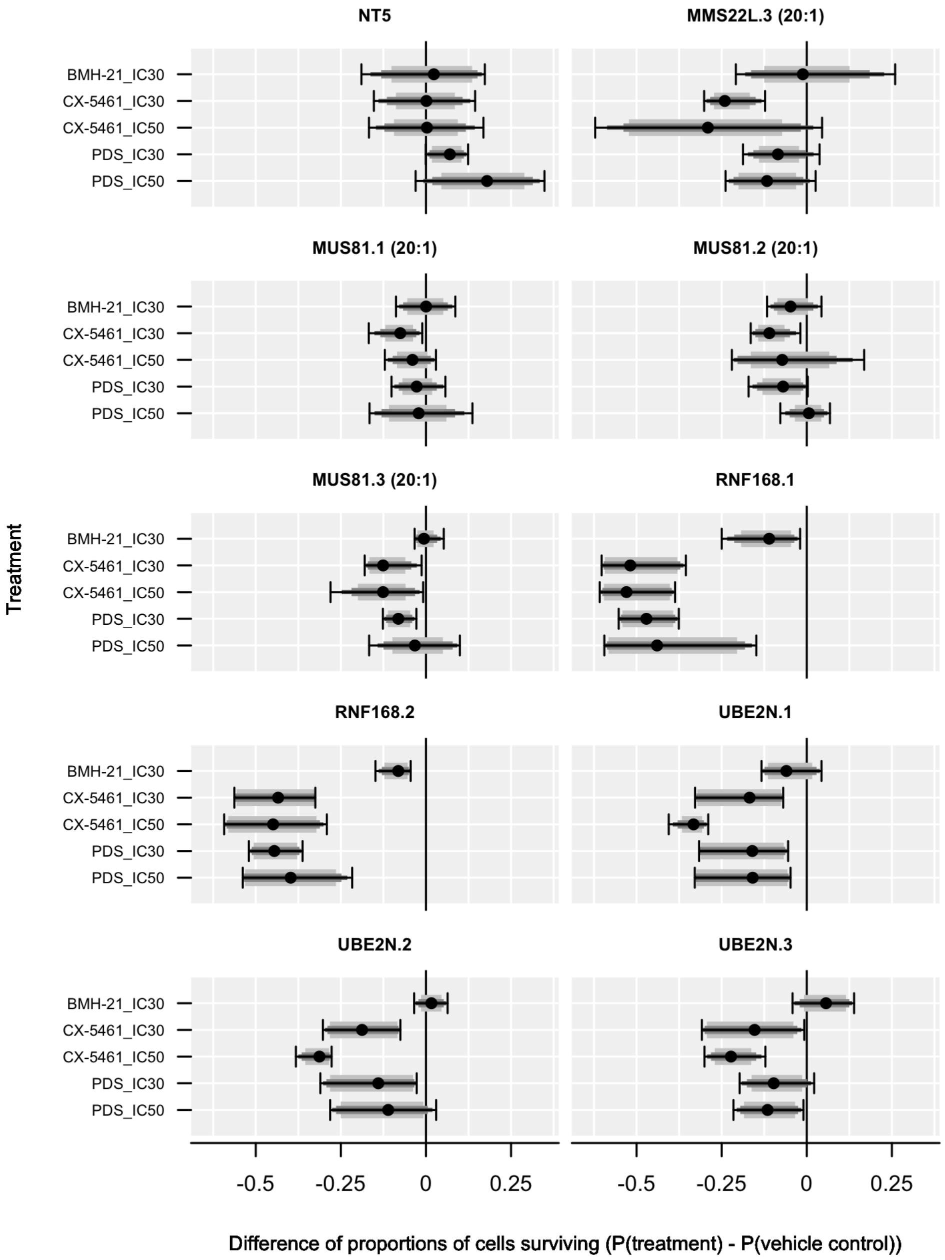

Supplementary Figure 9: Confidence intervals for CGA. For the CGA assay, statistical analysis was performed for each sgRNA by bootstrapping (see Methods). The x-axis indicates the confidence intervals and y-axis shows different treatment groups. The four confidence intervals are portrayed as: 95% CI as very wide light grey box (relative width 8), 99% CI as wide grey box (width 4), 99.9% CI as narrow dark grey box (width 2), and 99.99% CI as black line with end caps (width 1).

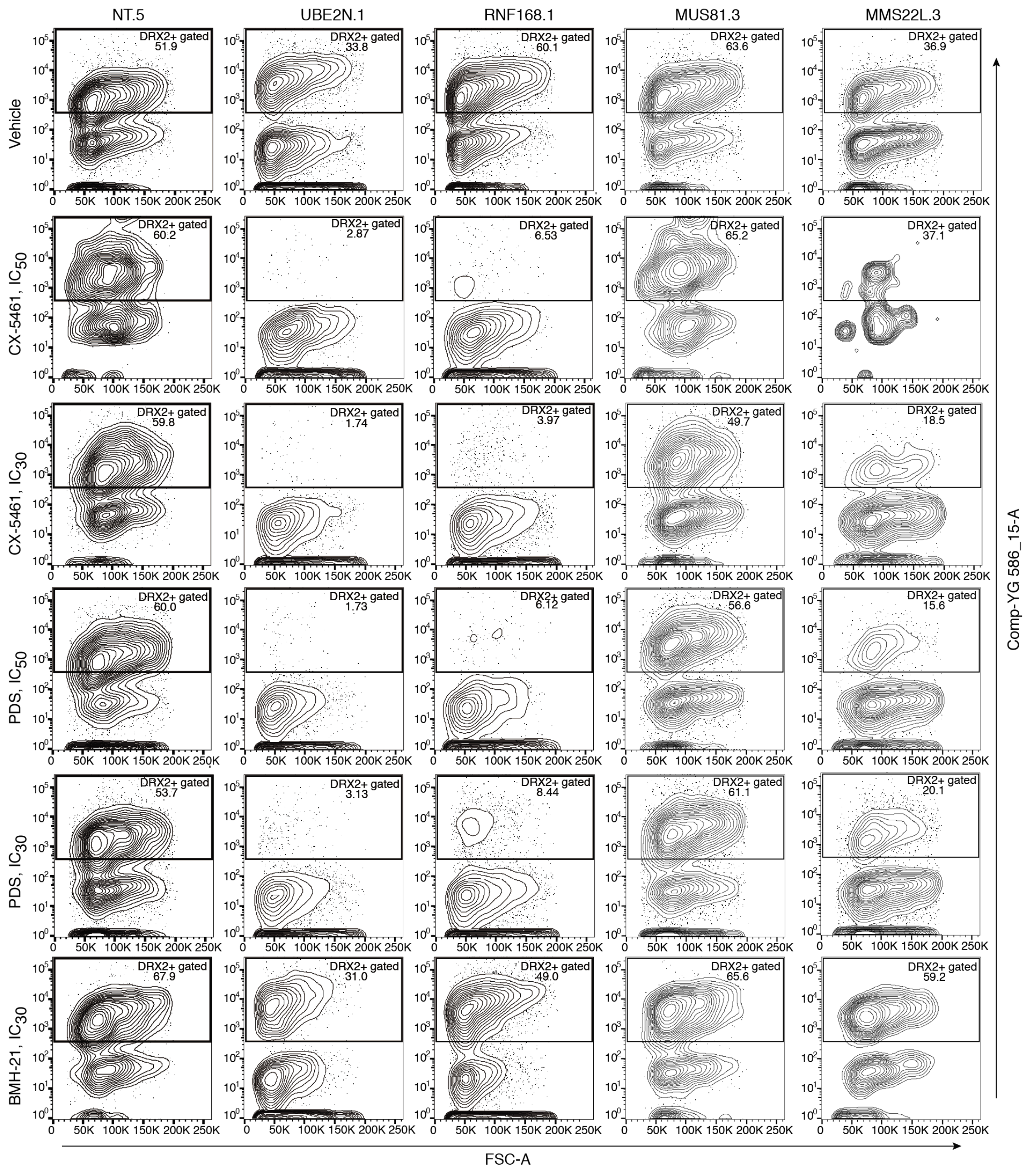

Supplementary Figure 10: Flow Cytometry Analysis for CGA.

Supplementary Figure 10: Flow Cytometry Analysis for CGA. HCT116 cells were transduced with individual sgRNAs including a non-targeting control (NT5), three targeting UBE2N, two for RNF168, three for MUS81, two for MMS22L for CGA as described above. Flow analysis was performed on cells transduced with the indicated sgRNA and treated with the indicated drugs to determine the fraction of cells expressing red fluorescence (DRX2+). The numbers within the charts indicate the percentage of the DRX2+ populations within the live cell population (gated on FSC-A and SSC-A). Data shown here are representative of one sgRNA for each gene from one experiment.

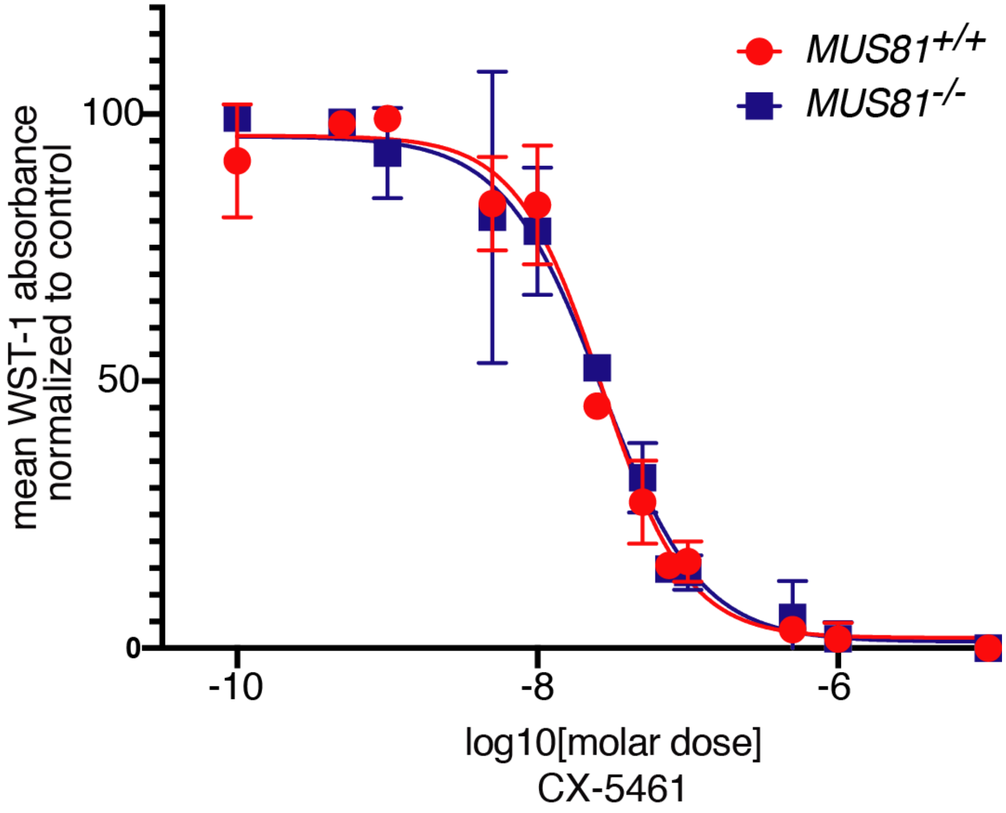

Supplementary Figure 11: CX-5461 drug dose response in MUS81-wildtype and -null cells. *MUS81*$^{+/+}$ and *MUS81*$^{-/-}$ HCT116 cells were treated with the vehicle or CX-5461 for 6 days and mean WST-1 absorbance was determined.

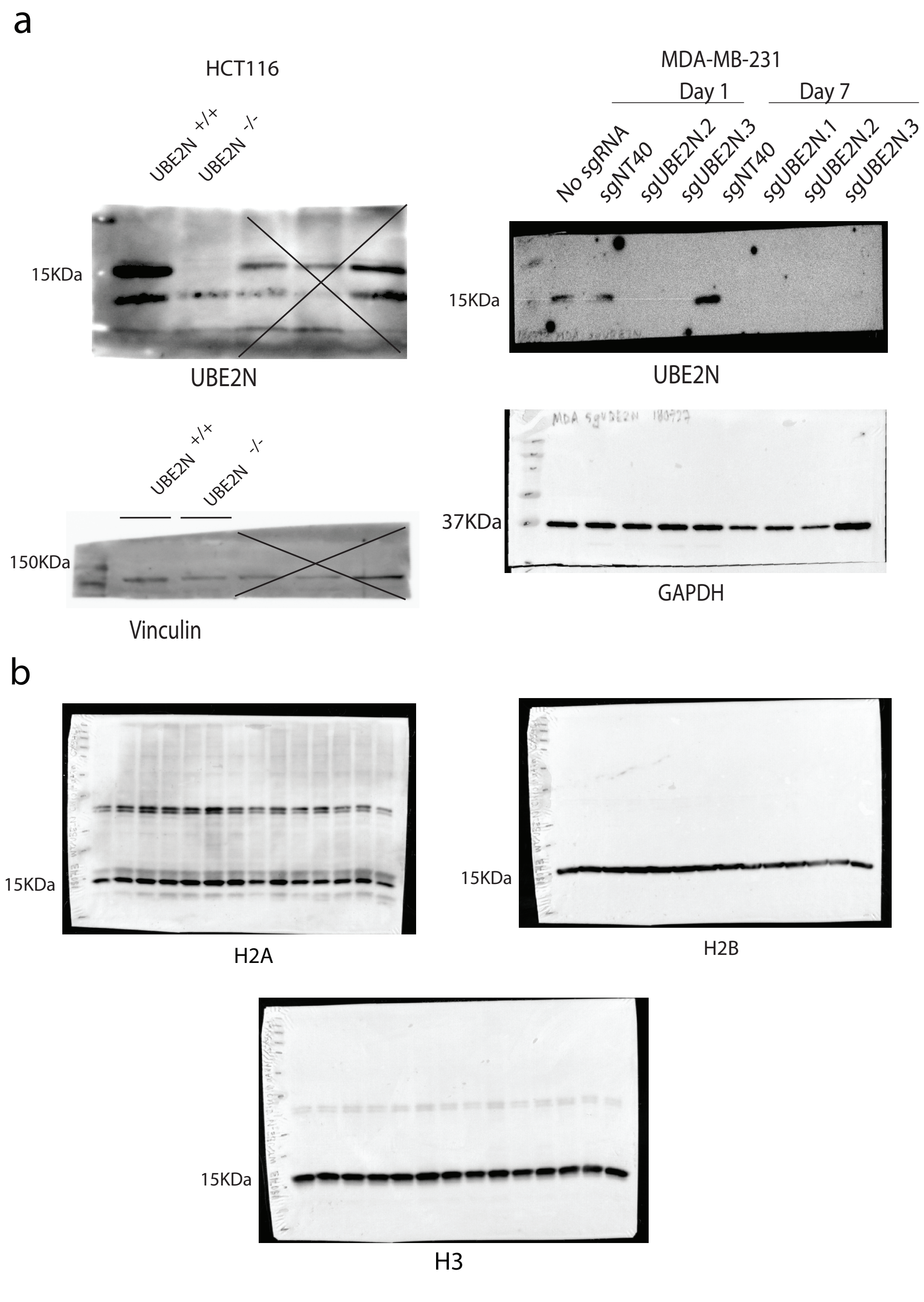

Supplementary Figure 12: Original Western blots for Figure 2f and Figure 4a.

(a) Original western blots for Figure 2f. Crossed lanes are irrelevant samples.

(b) Original western blots for H2A, H2B and H3 in Figure 4a

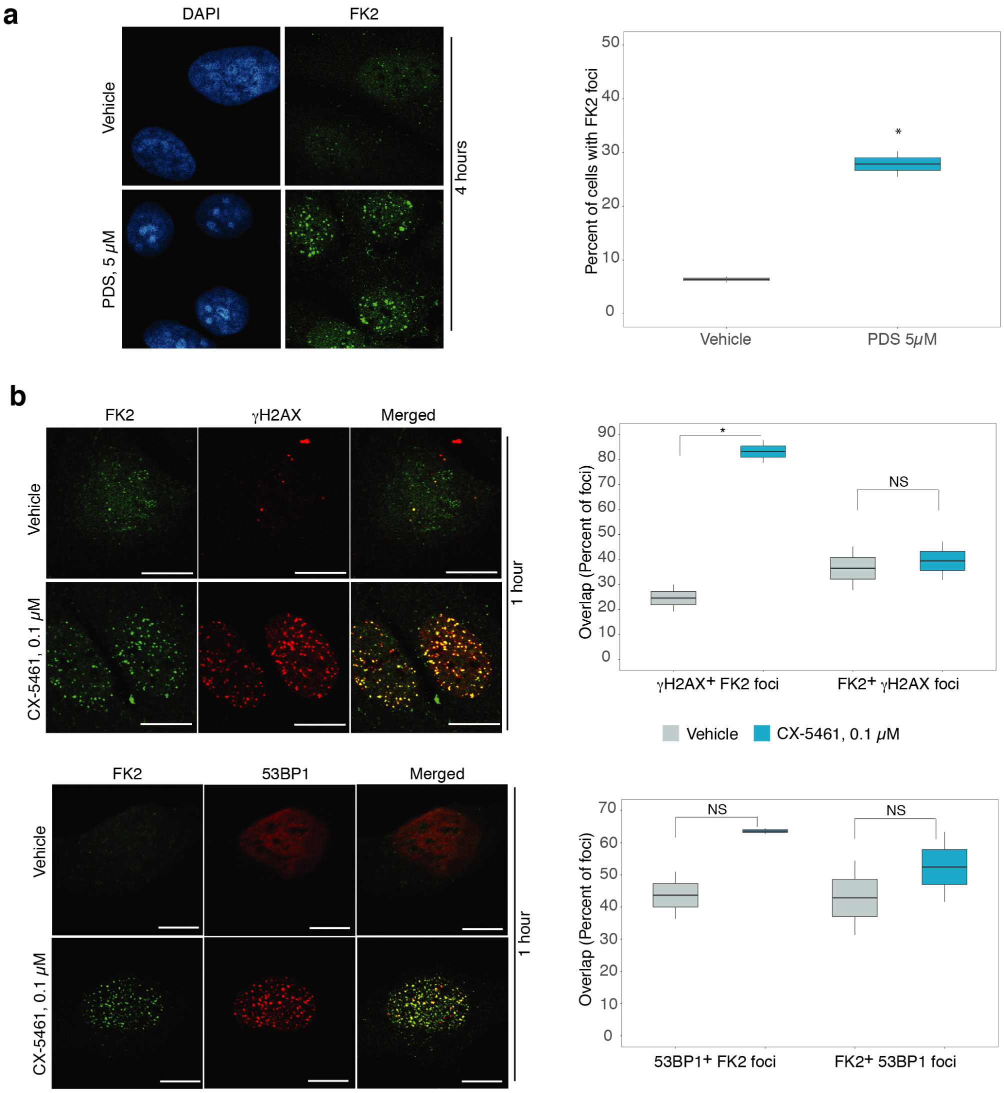

Supplementary Figure 13: CX-5461 induced FK2 foci.

Supplementary Figure 13: CX-5461 induced FK2 foci.

(a) FK2 foci were induced in U2OS cells after vehicle and PDS treatments at the indicated dose. Left panel shows the images of FK2 and DAPI staining. Right panel shows that the percent of cells with FK2 foci after CX-5461 is significantly higher than after vehicle treatment (* P-value$<$0.02, two-tailed unpaired t-test).

(b) FK2 foci co-localize with γH2AX (top panel) and 53BP1 (bottom panel) DNA damage foci after one hour of treatment with $0.1\,\mu$M CX-5461 in U2OS cells. The graph on the right shows the overlap of γH2AX /53BP1 foci and FK2 foci as either percent of total γH2AX/53BP1 foci that are also FK2 positive (γH2AX+ FK2 foci, 53BP1+FK2 foci) or percent of total FK2 foci that are also γH2AX/53BP1 positive (FK2+ γH2AX foci, FK2+53BP1 foci). * P-value$<$0.02, NS = not significant. Pictures are representative of at least three independent experiments. In each experiment, at least 100 cells were counted, and the experiments were repeated 3 or more times. All the data were pooled and statistical analysis (two-tailed unpaired t-test) was performed on all the pooled cells. Foci were counted by Spotcounting software developed by Steven Poon. The software is publicly available at github: https://github.com/shahcompbio/SpotCountingApp.Scale bars represent $10\,\mu$M.

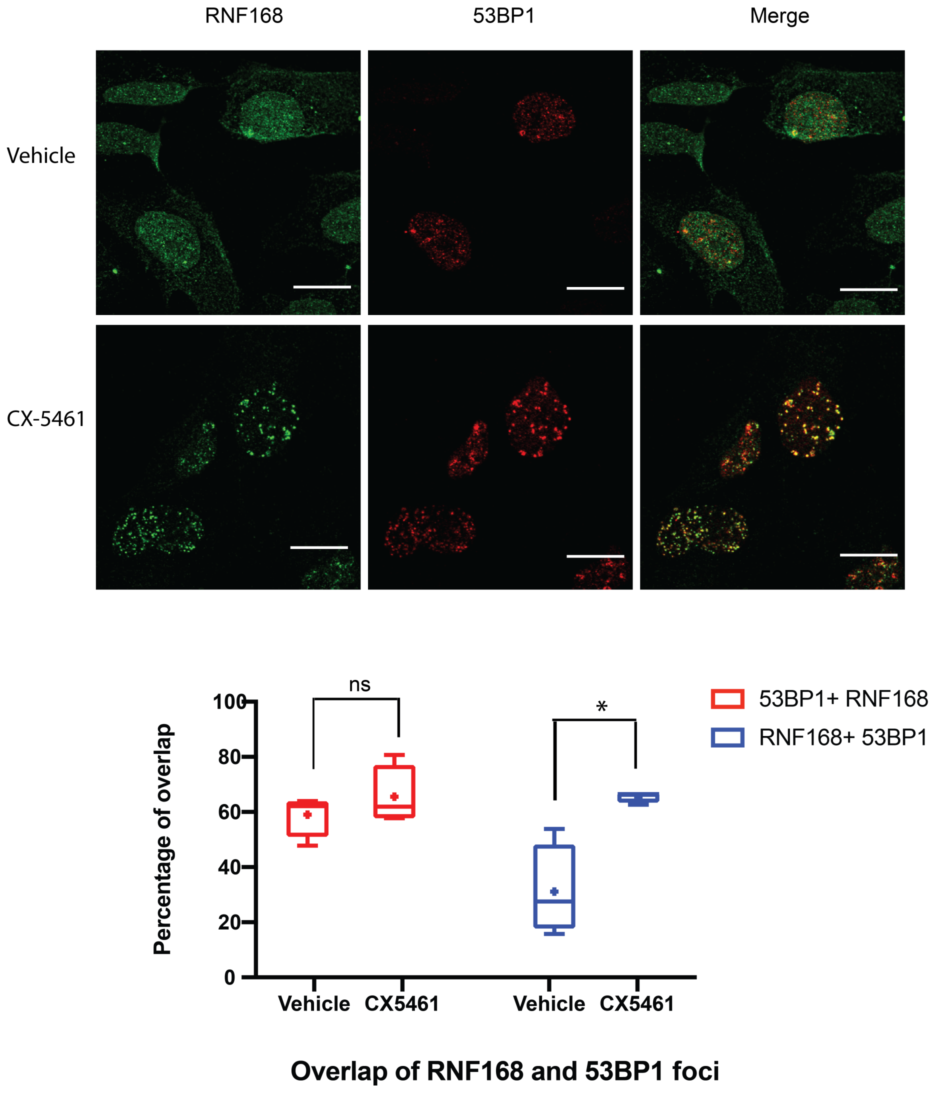

Supplementary Figure 14: RNF168 foci co-localize with 53BP1 after 4 hours of treatment with 1 μM CX-5461 in U2OS cells.

Supplementary Figure 14: RNF168 foci co-localize with 53BP1 after 4 hours of treatment with 1 µM CX-5461 in U2OS cells. Top panel shows the images of RNF168, 53BP1 and the co-staining. Bottom panel shows the overlap of RNF168 foci and 53BP1 foci as either percent of total 53BP1 foci that are also RNF168 positive (53BP1+ RNF168 foci) or percent of total RNF168 foci that are also 53BP1 positive (RNF168+ 53BP1). Foci were counted by Spotcounting software developed by Steven Poon. The software is publicly available at github: https://github.com/shahcompbio/SpotCountingApp. * P-value$<$0.05, NS = not significant. Box plots show the 10-90 percentile and medium. Means are shown as ”+”. The experiment were repeat at least 3 times and each time more than 50 cells in each condition were scored. Two-tailed student t-tests with unequal variance were performed. Scale bars in all experiments represent 10 µM.

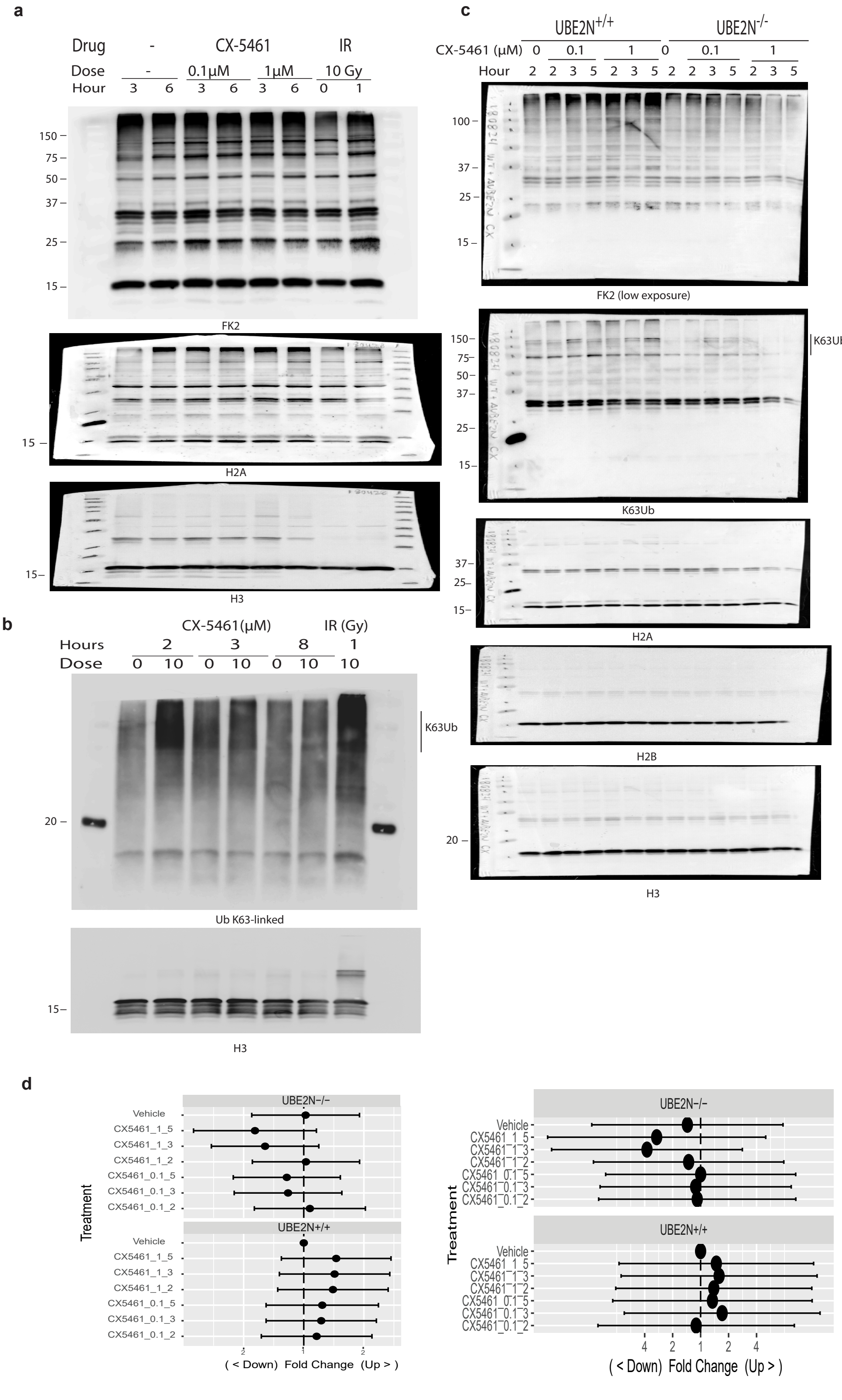

Supplementary Figure 15: CX-5461-induced chromatin ubiquitination

Supplementary Figure 15: CX-5461 induces DDR-associated chromatin ubiquitination. (a, b, c) *UBE2N*$^{+/+}$ **(a, b)** and *UBE2N*$^{-/-}$ **(c)** HCT116 cells were treated with either vehicle or CX-5461 at indicated doses and time periods, and chromatin fractions were extracted. Chromatin ubiquitination was examined using Western blots with antibodies against conjugated ubiquitin (FK2), K63-linked ubiquitin (K63Ub), histones H2A, H2B and H3. Molecular weight markers (KDa) are shown on the left.
(d) Confidence interval plots showing UBE2N-dependent histone ubiquitination at FK2 and K63 using antibodies specifically targeting FK2 or K53-linked ubiquitination in Western blot assays described in Figure 4a. Left panel shows FK2 and right panel shows K63Ub quantification. The signal intensity of antibodies in the western blot was quantified with Image J v1.51, which is publicly available at https://imagej.net/Download Total H2A measured via bands at 15kDa and around 30kDa (possibly dimers of H2A) provided a loading control. The dark circles indicate mean estimates of fold-change after drug treatment relative to the vehicle. The horizontal bars indicate 95% confidence intervals.Detailed statistical analysis was described in the Methods section.

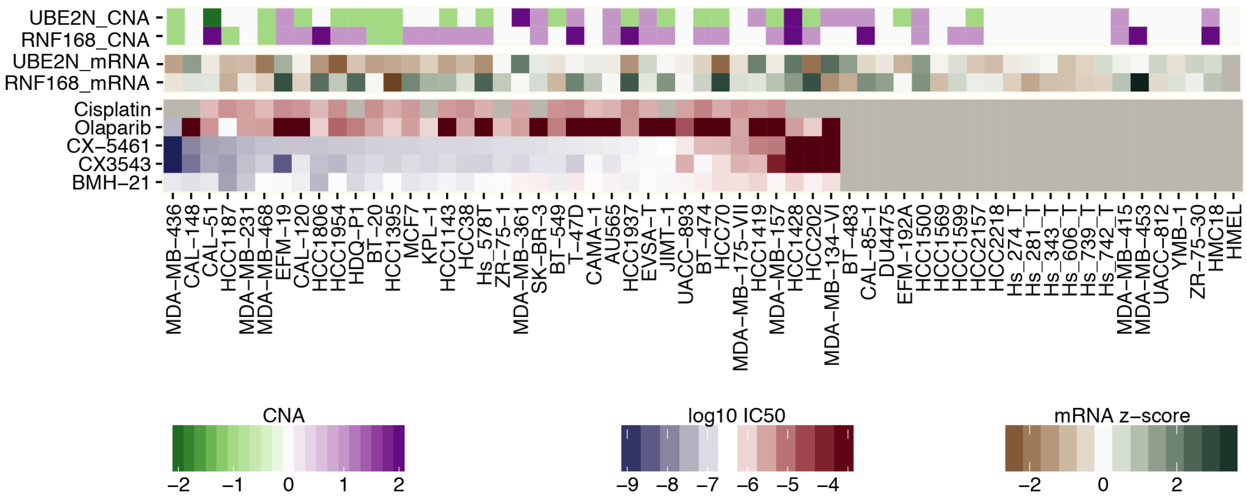

Supplementary Figure 16: UBE2N and RNF168 copy number alterations (CNA) and mRNA expression, and sensitivity of breast cancer cell lines to multiple drugs. Drug sensitivity data, represented as $\log_{10}$ IC_{50}, was previously published[27]. Gene copy number alterations (CNA) and mRNA (RNAseq) median z-score data was extracted from Cancer Cell Line Encyclopedia[100].

Supplementary Table 1: Comparison of top 10% depleted genes with Hart et al. fitness genes with Bayes factor (BF).

Gene	Rank	p-value	FDR	BF (HCT116)
Core fitness genes				
POLR2I	1	9.34E-06	0.002475	193.842
ANAPC5	3	8.41E-05	0.012871	220.05
RRM2	4	0.00010275	0.012871	52.962
SMC1A	5	0.00012143	0.012871	210.595
RAD9A	6	0.00028956	0.025578	32.268
PSMB5	8	0.00075659	0.050124	131.102
HAUS1	9	0.0015412	0.090759	55.665
TOP2A	10	0.0019895	0.105446	232.74
PSMD12	11	0.0027368	0.131863	-0.486
PSMB3	12	0.0043994	0.182815	114.009
DNA2	13	0.0045302	0.182815	241.739
PCNA	14	0.0048291	0.182815	377.499
H2AFX	15	0.0055016	0.194389	10.253
ANAPC4	18	0.0083038	0.23173	174.037
RBBP4	19	0.0088455	0.23173	43.178
POLR2D	20	0.0088642	0.23173	89.3
PSMB7	21	0.0091818	0.23173	251.667
PPP4C	22	0.010004	0.240999	-19.059
PSMD4	24	0.012787	0.273861	100.462
PNKP	25	0.012918	0.273861	101.705
RAD21	28	0.016337	0.309229	25.641
BARD1	29	0.017588	0.321441	128.903
CDC20	30	0.02082	0.356915	276.737

Continued on next page

Gene	Rank	p-value	FDR	BF (HCT116)
CDC23	31	0.020876	0.356915	79.064
PSMC4	32	0.023137	0.383199	48.627
PSMD7	33	0.025939	0.409351	58.496
PLK1	34	0.026985	0.409351	327.51
GINS3	36	0.028087	0.409351	209.274
CDC45	37	0.028647	0.409351	133.704
PSMB1	38	0.029563	0.409351	249.359
CDC16	39	0.032178	0.409351	332.911
POLE2	40	0.032888	0.409351	325.426
PKMYT1	41	0.032907	0.409351	99.95
VCP	42	0.03315	0.409351	338.776
WEE1	44	0.034532	0.409351	148.976
PSMA7	45	0.034756	0.409351	187.614
RUVBL1	47	0.042247	0.462821	261.575
POLR2E	48	0.042509	0.462821	340.633
HCT116-dependent fitness genes				
GAPDH	2	9.34E-06	0.002475	11.088
MAD2L1	23	0.0109	0.251184	31.295
RBX1	26	0.013889	0.28313	8.352
XRCC6	35	0.027956	0.409351	13.343
PSMC1	43	0.033224	0.409351	68.852
CDC7	46	0.039613	0.456414	147.986

Supplementary Table 2: sgRNA sequences used for genetic validation.

Gene	sgRNA ID	Source	Sequence
MMS22L	sgMMS22L.1	Deskgen	CCAGCCAATGTATATCCAAG
MMS22L	sgMMS22L.2	Library	GCTTAAGGGCTCCGCTGCAG
MMS22L	sgMMS22L.3	Deskgen	AAAGCCTCATAAGGATGGAC
MUS81	sgMUS81.1	Deskgen	AGGACTCTTCCATGCCAGTG
MUS81	sgMUS81.2	Deskgen	GGTTCCTGCCCAGCCCAAAG
MUS81	sgMUS81.3	Deskgen	ACTTCCGCAAGTCGCCCCTG
Non-targeting	sgNT5	GeCKOv2	GGAGGGATTGGGAGCTTGACA
Non-targeting	sgNT40	Library	ACATAGTCGACGGCTCGATT
RNF168	sgRNF168.1	Deskgen	GAGTCCACGACGATACCCGG
RNF168	sgRNF168.2	Deskgen	AAGAAATTCTCTCGTCAACG
RNF168	sgRNF168.3	Deskgen	GATCTGCATGGAAATCCTCG
UBE2N	sgUBE2N.1	Library	CGGTTACCTTGATGATCCTG
UBE2N	sgUBE2N.2	Deskgen	GATGATCCTGCGGGGCAGCC
UBE2N	sgUBE2N.3	Library	ATAGGAAACCCAGCGTTTGC

Supplementary Table 3: Antibodies used for Western blots.

Antibody	Company	Catalogue number	Dilution
GAPDH	Santa Cruz	sc-48166	1:1000
GAPDH	Millipore Sigma	MAB374	1:5000
H2A	Abcam	ab18255	1:1000
H2B	Abcam	ab1790	1:30000
H3	Abcam	ab1791	1:30000
RNF168	Abcam	ab229271	1:1000
UBE2N	ThermoFisher Scientific	37-1100	1:2000

Continued on next page

Supplementary Table 3 – *Continued from previous page*

Antibody	Company	Catalogue number	Dilution
FK2	Enzo Life Sciences	BML-PW8810-0100	1:500
Ubiquitin, linkage-specific K63	Abcam	ab179434	1:1000
Vinculin	Sigma	V9131	1:10000
